# Altered serum amyloid beta and cerebral perfusion and their associations with cognitive function in patients with subcortical ischemic vascular disease

**DOI:** 10.3389/fnins.2022.993767

**Published:** 2022-10-13

**Authors:** Wei Zhang, Mingxu Li, Xia Zhou, Chaojuan Huang, Ke Wan, Chenchen Li, Jiabin Yin, Wenming Zhao, Cun Zhang, Xiaoqun Zhu, Zhongwu Sun

**Affiliations:** ^1^Department of Neurology, First Affiliated Hospital of Anhui Medical University, Hefei, China; ^2^Department of Radiology, First Affiliated Hospital of Anhui Medical University, Hefei, China

**Keywords:** arterial spin labeling, biomarker, cognitive impairment, subcortical ischemic vascular disease, serum amyloid beta

## Abstract

Subcortical ischemic vascular disease (SIVD) is one of the important causes of cognitive dysfunction, altered amyloid-beta (Aβ) and cerebral perfusion may be involved in the pathophysiological mechanism of SIVD and are closely related to cognitive function. We aimed to investigate altered serum Aβ and cerebral perfusion in patients with SIVD and their correlation with cognitive function. Seventy-four healthy controls (HCs) and 74 SIVD patients, including 38 SIVD patients with no cognitive impairment (SIVD-NCI) and 36 SIVD patients with mild cognitive impairment (SIVD-MCI) underwent the measurement of serum Aβ40 and Aβ42 levels, pseudo-continuous arterial spin labeling MRI scanning, and cognitive evaluation. Compared to the healthy controls (HCs), the level of serum Aβ40 and Aβ40/42 ratio increased and Aβ42 decreased in SIVD patients. The serum Aβ40 level and Aβ40/42 ratio in patients with SIVD-MCI were significantly higher than those in the HCs and SIVD-NCI, and the level of Aβ42 in the SIVD-MCI was lower than the HCs. In addition, the serum Aβ40/42 ratio provided high diagnostic accuracy for SIVD and SIVD-MCI, it was further identified as an independent risk factor for cognitive impairment. Patients with SIVD-NCI and SIVD-MCI exhibited both increased and decreased cerebral blood flow (CBF) in regional. The Aβ40/42 ratio was associated with global CBF, while altered global and regional CBF was associated with cognitive deficits. In addition, white matter hyperintensities volume (WMHV) correlated with Aβ40/42 ratio, CBF, and cognition. The relationship between Aβ40/42 ratio and cognition was partially mediated by altered CBF. Based on these results, we conclude that the serum Aβ40/42 ratio may be a potential biomarker that can complement current methods for the prediction and diagnosis of cognitive impairment in SIVD patients. In addition, serum Aβ may play a role in cognitive function by regulating CBF, which provides new insights into the intervention, treatment, and prevention of cognitive impairment in SIVD.

## Introduction

Nowadays, the pathogenesis of subcortical ischemic vascular disease (SIVD) is not well defined. The term SIVD refers to a subtype of vascular cognitive impairment (VCI) characterized by extensive white matter hyperintensities (WMHs) and multiple lacunar infarcts in brain imaging (Jokinen et al., 2006). The symptoms of SIVD are usually not evident in the early stages, but such individuals may develop cognitive deficits in the future (Sachdev et al., 2014). Patients with SIVD are susceptible to decreased cerebral perfusion, which could lead to cognitive impairment including speed of information processing, executive function, and attention (Galluzzi et al., 2005; Sun et al., 2016). Amyloid beta (Aβ) accumulation in the brain is a characteristic symptom of Alzheimer’s disease (AD), recent studies showed that approximately 30% of patients with clinically diagnosed subcortical vascular cognitive impairment (SVCI) had a significant amyloid load (Lee et al., 2011). However, the role of Aβ in cognitive impairment in SIVD patients and its relationship with cerebral perfusion are largely unknown.

With the development of neuroimaging technology, we can better explore the underlying pathogenesis of cognitive impairment in SIVD patients with the help of brain magnetic resonance. Arterial spin labeling (ASL) MRI employs an in vivo technique to noninvasively quantify cerebral blood flow (CBF) by using arterial water protons as an endogenous tracer (Inoue et al., 2014). A previous study found that compared with SIVD patients without cognitive impairment, SIVD patients with cognitive impairment showed a diffuse decrease in brain CBF, and regional CBF values were positively correlated with cognitive scores (Sun et al., 2016). In addition, CBF in the frontal and parietal cortices was significantly lower in patients with SVCI and was not associated with brain atrophy (Schuff et al., 2009). Studies have shown that CBF in the normally occurring WM region around WMHs had begun to decline and may progress to WMHs (Wu et al., 2019), so it was speculated that regional CBF changes may be an earlier danger signal and an initiating factor in the pathogenesis of SIVD and cognitive impairment.

Deposition of Aβ within small cerebral vessels is a pathological feature of cerebral amyloid angiopathy (CAA). Growing evidence suggests that CAA plays a key role in the pathogenesis of dementia (Keage et al., 2009). Aβ is produced by the sequential processing of amyloid precursor protein by β-secretase and ɣ-secretase, and a peptide consisting of 40 or 42 amino acids is the main product (Aβ40 and Aβ42, respectively) (Gandy, 2005). Aβ40 is the main component of AD brain amyloid plaques and Aβ42 is the predominant component of vascular deposits in CAA (Yamada, 2015). A Vilar-Bergua et al. (2016) conducted a summary analysis of SIVD cerebrospinal fluid (CSF) biomarkers and found that Aβ42 could effectively distinguish vascular dementia (VaD) from AD and healthy control (HC), in addition, plasma Aβ42 was also one of the best biomarkers for silent brain infarct and WMHs. Due to the traumatic nature of CSF examination, many studies have been devoted to exploring whether blood Aβ markers have similar diagnostic values in recent years. A blood-based test for the differential diagnosis of VaD and other types of dementia found that plasma Aβ40 was significantly elevated and Aβ38/Aβ40 ratio was decreased in VaD patients, moreover, the diagnostic accuracy of plasma Aβ38/Aβ40 ratio for VaD can be comparable to that of CSF biomarkers (Bibl et al., 2007). However, there are few studies on the role of Aβ40 and Aβ42 in SIVD and its cognitive impairment.

It is now recognized that both SVCI and AD are susceptible to vascular risk factors and can be secondary to chronic cerebral hypoperfusion affecting cognitive function (Gorelick et al., 2011; Snyder et al., 2015). Some scholars put forward the hypothesis that AD may be of vascular origin 25 years ago (de la Torre and Mussivand, 1993), an important reason for the accumulation of Aβ in brain tissue is the decreased clearance, and the normal clearance of Aβ depends on stable CBF (Tarasoff-Conway et al., 2015). Studies have confirmed that disordered CBF can lead to decreased cerebral perfusion, damage to the blood-brain barrier, and cause Aβ clearance obstacles, resulting in more Aβ deposition in brain tissue (Bell and Zlokovic, 2009). Recently, it has even been shown that the earliest event in AD is CBF reduction, which subsequently may play a key role in driving cognitive decline by initiating the amyloid cascade itself or by causing and amplifying Aβ production (Korte et al., 2020). However, on the other hand, Aβ deposited in blood vessels has certain toxic effects, which may further lead to hypoperfusion by inducing vasoconstriction, increasing vascular resistance, and causing pericyte degeneration (Kisler et al., 2017; Yew and Nation, 2017). Thus, the causal relationship between hypoperfusion and Aβ accumulation is still unknown. Although CBF and Aβ play crucial roles in cognitive impairment in SIVD patients, few studies have explored the relationship between CBF and Aβ in SIVD patients with cognitive impairment and how the causal relationship affects cognitive function.

In this study, we performed serum Aβ, cognitive function, and cerebral perfusion imaging analysis in SIVD patients to test the following hypotheses. Firstly, SIVD patients (including SIVD-NCI and SIVD-MCI) showed significant changes in serum Aβ levels, which may be an independent risk factor for cognitive impairment. Secondly, patients with SIVD showed decreased global cerebral perfusion, which was related to Aβ levels, and regional cerebral perfusion changes were associated with specific cognition domains. Finally, altered CBF mediated the link between serum Aβ levels and cognitive impairment in SIVD patients.

## Materials and methods

### Participants

This cross-sectional study was approved by the Ethics Committee of The First Affiliated Hospital of Anhui Medical University, and the study conformed to the World Medical Association Declaration of Helsinki. All participants signed an informed consent form before participating. In this study, from August 2020 to April 2022, 120 patients with SIVD were included from the Department of Neurology of the First Affiliated Hospital of Anhui Medical University, Hefei, China. Among the 120 participants, we excluded seven patients with cortical non-lacunar infarcts and five with non-vascular white matter hyperintensities. Others with intracerebral hemorrhages (*n* = 3), cardioembolic infarction (*n* = 2), vascular dementia (*n* = 4), Alzheimer’s disease (*n* = 3), Parkinson’s disease (*n* = 3), new subcortical infarction (*n* = 3), subcortical ischemic depression (*n* = 4), taking cognitive-improving drugs (*n* = 3), alcohol abuse (*n* = 2), severe hepatic and renal insufficiency (*n* = 2), inability to complete neuropsychological tests (*n* = 2), claustrophobia (*n* = 3). Hence, a final analysis of 74 SIVD-eligible patients was performed, including 38 SIVD patients with no cognitive impairment (SIVD-NCI) and 36 SIVD patients with mild cognitive impairment (SIVD-MCI). All magnetic resonance imaging was evaluated in a standardized manner by two experienced neuroradiologists, and they were blinded to the clinical data of the subjects in advance. The inclusion criteria for patients with SIVD were based on the diagnostic criteria proposed by Roman et al. (2002) and revised as follows: (1) age between 50 and 80 years; (2) brain MRI showed extensive periventricular and deep white matter hyperintensity; extensive cap-shaped hyperintensity (>10 mm along the ventricle) or irregular band-shaped hyperintensity (wide degree > 10 mm, irregular borders and extending into deep white matter); and diffuse confluent hyperintensity (>25 mm, irregular shape, diffuse hyperintensity without focal lesions); and lacune(s) in the deep gray matter; or (3) multiple lacunes (>5) in the deep gray matter and accompanied by moderate white matter hyperintensities; and (4) absence of hemorrhages, cortical and/or corticosubcortical non-lacunar territorial infarcts and watershed infarcts; signs of normal pressure hydrocephalus; and specific causes of white-matter lesions. In addition to meeting the above imaging standards, SIVD-MCI patients should also meet the following points: (1) participants complained of cognitive decline, and this performance lasted at least several months to several years; (2) in the cognitive test, at least one or more cognitive domains were impaired but did not reach the level of dementia (compared with the healthy controls, the composite *z*-score of at least one cognitive domain was lower than the adjusted average of 1.5 SD); (3) the ability of daily living was unaffected, and only complex activities were impaired or defective. Therefore, patients with SIVD-NCI meet the diagnostic criteria for SIVD and have normal cognitive function and activities of daily living. Subjects meeting the following criteria will be excluded: (1) history of traumatic brain injury, cerebral hemorrhage, intracranial tumor, serious physical disease, mental system disease, or history of electroconvulsive therapy; (2) conventional magnetic resonance imaging showed infarction with a diameter ≥ 15 mm; (3) epilepsy, alcoholism, Parkinson’s disease, Alzheimer’s disease, dementia with Lewy bodies and other diseases that may cause cognitive impairment; (4) white matter lesions caused by other causes, such as normal pressure hydrocephalus, multiple sclerosis, brain radiation, and metabolic disease; (5) Hamilton Depression Scale score > 17, or anxiety; (6) severe visual or hearing loss cannot complete neuropsychological evaluation; (7) MRI examination is contraindicated or cannot tolerate MRI examination.

In this study, 74 age, sex, and education-matched healthy controls (HCs) were recruited from the local community during the same period and did not meet the inclusion criteria of the SIVD patients.

### Neuropsychological test

The neuropsychological tests of this research were uniformly evaluated by doctoral students in neurology who had undergone rigorous training. All subjects participated in our cognitive tests to fully understand their health status, which ensured the compliance of the cognition test and the reliability of the results. All participants underwent the following neuropsychological assessments: (1) life abilities evaluation: Activities of Daily Living scale (ADL); (2) global cognition test: Mini-Mental State Examination (MMSE); (3) information processing speed test: Trail Making Test-A (TMT-A), the Stroop’s Color Word Test-A (SCWT-A: dot), the Stroop’s Color Word Test-B (SCWT-B: word), and the Symbol Digit Modalities Test (SDMT); (4) executive function: Trail Making Test-B (TMT-B) and Stroop’s Color Word Test-C (SCWT-C: color word); (5) attention function: The forward and backward aspects of the Digital Span test (DS); (6) memory function: Auditory Verbal Learning Test (AVLT), and consists of four parts: immediate memory, 5-min delay memory, 20-min delay memory, and recognition memory. (7) language function: Verbal Fluency Test (VFT); (8) visuospatial perception test: Clock Drawing Test (CDT). (9) Hamilton Depression (HAMD) scale was assessed to exclude those with potential depression. To facilitate a better comparison of cognitive function between groups, we performed z-transformation on the neuropsychological test of each subject (*z*-score = individual test score minus mean of healthy controls, divided by standard deviation of controls). The subitems of each cognitive domain were averaged to obtain the composite score of a single domain (The *z*-score of the SDMT item was first multiplied by –1, then averaged with STROOP-A and STROOP-B to obtain the *z*-score of the cognitive domain of information processing speed. Subitems in other cognitive domains have not been treated similarly). It is well known that the higher the score of the information processing speed and executive function is, the worse the cognitive performance; therefore, we invert the *z*-scores of the two parts by multiplying them by –1, and higher *z*-scores represent better performance. In addition, higher *z*-scores in language, memory, attention, and visuospatial domains indicate better cognitive function.

### Clinical and biochemical assessments

Demographic and clinical data of all subjects were collected, including sex, age, education level; history of stroke, hypertension, diabetes, hyperlipidemia, drinking, and smoking; medical history related to Aβ40 or Aβ42, and vascular risk factors. In addition, detailed neurological physical examination and clinical interview were performed on all subjects by two neurologists. Subjects fasted for more than 12 h. On the day of performing the brain MRI, 3 ml of peripheral venous blood was drawn on an empty stomach in the morning and immediately sent to the laboratory of the First Affiliated Hospital of Anhui Medical University to complete liver and kidney function, triglycerides (TG), total cholesterol (TCH), high-density lipoprotein-cholesterol (HDL-C), low-density lipoprotein-cholesterol (LDL-C), homocysteine (Hcy), and hs-CRP detection. Additionally, 2 ml of peripheral venous blood was taken at 3000 rpm/separation of the heart for 20 min, the serum was taken in the Eppendorf tube and placed in the refrigerator at –80°C until it was used for testing. Using a two-site sandwich enzyme-linked immune sorbent assay to determine serum Aβ40 and Aβ42 levels (Huada Biotech Corporation Ltd., Wuhan, China). The dynamic range was 8–500 pg/ml for Aβ40 and 2–85 pg/ml for Aβ42. The intraassay and interassay coefficients of variation for serum Aβ40 and Aβ42 levels were less than 9 and 11%, respectively.

### APOE genotyping

APOE genotyping was performed on EDTA blood samples at Beijing Liuhe Huada Gene Technology company. Genomic DNA was extracted from blood samples using the Tiangen Biochemical Technology company Blood DNA Mini Kit (DP348). Genotyping of rs429358 and rs7412 (APOE) in each subject was performed using the Penta-primer amplification refractory mutation system method. After the polymerase chain reaction, the plates were read by a TECAN M1000 infinite reader, and DNA sequences were analyzed using the online software snp decoder1. The χ^2^ test was used to assess whether the allele frequency was consistent with expectation in the Hardy–Weinberg equilibrium. The statistical significance level was set at *P* < 0.05.

### Image acquisition

MRI scans were obtained using a 3.0-Tesla MR system (Discovery MR750w, General Electric, Milwaukee, WI, USA) with a 24-channel head coil. Earplugs were used to reduce scanner noise, and tight but comfortable foam padding was used to minimize head motion. High-resolution 3D T1-weighted structural images were acquired by employing a brain volume (BRAVO) sequence with the following parameters: repetition time (TR) = 8.5 ms; echo time (TE) = 3.2 ms; inversion time (TI) = 450 ms; flip angle = 12°; field of view (FOV) = 256 mm × 256 mm; matrix size = 256 × 256; slice thickness = 1 mm, no gap; 188 sagittal slices. T2 fluid-attenuated inversion recovery (FLAIR): TR = 9,000 ms, TE = 119.84 ms, FOV = 225 × 225 mm, FA = 160°, Matrix = 512 × 512, number of layers = 19, layer thickness = 7 mm, acquisition time = 1 min 57 s. SWAN: TR = 45.4 ms, TE = 23.536 ms, FOV = 240.64 × 240.64 mm, FA = 20°, Matrix = 512 × 512, number of layers = 138, layer thickness = 1 mm, acquisition time = 3 min and 51 s. Resting-state perfusion imaging was performed using a pseudo-continuous arterial spin labeling (ASL) sequence with a 3D fast spin-echo acquisition and background suppression (TR = 5070 ms, TE = 11.5 ms; post-label delay = 2025 ms; spiral in readout of eight arms with 512 sample points; flip angle = 111?; FOV = 240 mm × 240 mm; reconstruction matrix = 128 × 128; slice thickness = 3 mm, no gap; 50 axial slices; number of excitation = 3). The label and control whole-brain image volumes required 8 TRs, respectively. Routine T2-weighted images were also collected to exclude any organic brain abnormality. None of the participants were excluded for visually inspected imaging artifacts.

### Structural MRI data processing

The three-dimensional (3D) structural MRI images were processed using the Computational Anatomy Toolbox 12 (CAT12) software^1^ based on SPM8. Firstly, all the structural images were corrected for bias-field inhomogeneities. Then, the structural images were segmented into three density components including gray matter (GM), white matter (WM), and cerebrospinal fluid (CSF). Total brain volume (TBV) was calculated as the sum of total GM and WM. Intracranial volume (ICV) was the summation of all tissue classes (total GM, total WM, and CSF volume). The whole white matter hyperintensities volume (WMHV) was segmented by the lesion prediction algorithm using T2 fluid-attenuated inversion recovery imaging (FLAIR) as implemented in the Lesion Segmentation Toolbox (LST) version 3.0.0^2^ for SPM, and volumes of total WMH were estimated as direct volumes in ml.

### PCASL data processing

Three ASL difference images were calculated by subtracting the label images from the control images and then averaged. Next, CBF was quantified by applying a single-compartment model (Buxton et al., 1998) to the mean ASL difference and proton-density-weighted reference images (Xu et al., 2010; Zhu et al., 2015, 2017). SPM12 software^3^ was used to normalize the CBF images into the MNI space using the following steps: (1) individual structural images were firstly co-registered with the CBF images; (2) the transformed structural images were segmented and normalized to the MNI space; and (3) the CBF image of each subject was written into the MNI space using the deformation parameter derived from the prior step and was resliced into a 2-mm cubic voxel. For standardization, the CBF value of each voxel was divided by the global mean CBF value of each person. Finally, the CBF images were smoothed with a 6 mm FWHM Gaussian kernel. Multiple comparisons were corrected using the cluster-level false discovery rate (FDR) method, resulting in a cluster defining threshold of *P* < 0.001 and a corrected cluster significance of *P* < 0.05 (Mueller et al., 2017). Clusters were localized by non-linear transformation to the anatomical automatic labeling (AAL) template provided by the Montreal Institute of Neuroscience. The results were presented using MRIcron^4^. The CBF value of regions with differing perfusion was extracted for further statistical analysis.

### Statistical analysis

The Shapiro–Wilk (S–W) test was performed on all continuous variables to assess whether the data conformed to normality. Normally distributed continuous variables were presented as mean ± standard deviation (SD), non-normally distributed continuous variables are presented as median with interquartile range (IQR), and values for categorical variables were provided as frequencies with percentages (%). A two-sample *t*-test was used for continuous variables with normal distribution, the Mann–Whitney *U* test was used for continuous variables with non-normal distribution, and a chi-square test was used for categorical variables between the comparison of the HC and SIVD groups. The comparison of continuous variables with normal distributions between the HC, SIVD-NCI, and SIVD-MCI groups used a one-way analysis of variance (ANOVA) followed by a post hoc Bonferroni test. Continuous variables with non-normal distributions were analyzed using Kruskal–Wallis tests, categorical variables were tested by chi-square tests, and the statistical significance of the pairwise comparison among the three groups of HC, CSVD-NCI, and CSVD-MCI was corrected by the Bonferroni method to control for false positives (*P* < 0.05/3 = 0.0167). To evaluate the diagnostic value of serum level of Aβ in distinguishing HCs from SIVD, and SIVD-NCI from SIVD-MCI, receiver operating characteristic (ROC) analysis was performed, and the area under the curve (AUC) was calculated to provide better tools for early diagnosis. Binary logistic regression analysis was used to investigate independent risk factors for SIVD-MCI, we took the occurrence of SIVD-MCI in SIVD patients as the dependent variable and included the influencing factors of *P* < 0.2 in the univariate logistic regression analysis as the independent variable Partial correlation analysis (controlling for age, gender, education, vascular risk factors) was used to analyze the correlation between the CBF (including the whole brain, GM, and WM), Aβ40/42 ratio, and WMHV. At the same time, a partial correlation analysis (controlling for age, gender, education, vascular risk factors) was carried out to explore the correlation between CBF, WMHV and cognitive function. After the CBF values of HC, SIVD-NCI, and SIVD-MCI groups were extracted, the differences between the two groups were compared by a two-sample *T*-test and corrected by the Bonferroni method (*P* < 0.05/3 = 0.0167) was considered statistically.

### Mediation analysis

To test whether the alteration of the CBF (including the whole brain and GM) mediated the link between the Aβ40/42 ratio and cognitive function, mediation analysis of linear regression models with the help of the PROCESS macro^5^ was performed, and a multifunctional modeling tool developed by Hayes in 2009 that can be used for SPSS analysis. This PROCESS used the common least squares path analysis framework to estimate the direct and indirect mediation effects. In the schematic diagram of the mediation analysis model (Figure 6A), all paths were reported as nonstandard ordinary least squares regression coefficients. Gender, age and years of education were included in the model as covariates. During the implementation of SPSS, significance analysis was performed based on 5,000 bootstraps, but the significance of the indirect effect needed to be evaluated by the bootstrap 95% confidence interval (CI). A significant indirect effect was considered when the 95% CI did not contain zero. The level of significance was set at *P* < 0.05.

## Results

### Participant characteristics, neuropsychological tests, and neuroimaging manifestations

**FIGURE 1:**
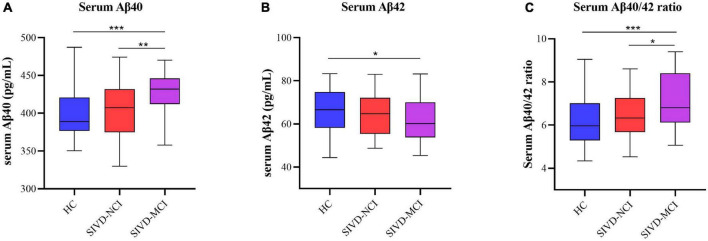
Boxplots of differences in serum Aβ40, Aβ42, and Aβ40/42 levels between HC, SIVD-NCI, and SIVD-MCI groups. **(A)** Comparison of serum Aβ40 levels among the three groups of HC, SIVD-NCI and SIVD-MCI. **(B)** Comparison of serum Aβ42 levels among the three groups of HC, SIVD-NCI and SIVD-MCI. **(C)** Comparison of serum Aβ40/42 levels among the three groups of HC, SIVD-NCI and SIVD-MCI. HC, healthy control; SIVD, subcortical ischemic vascular disease; NCI, no cognitive impairmen; MCI, mild cognitive impairment; *P < 0.05; **P < 0.01; ***P < 0.001.

The demographic, clinical data, neuropsychological tests, and neuroimaging manifestations for each group were listed in Table 1. No significant differences in age, gender, and educational levels were observed between the HC and whole SIVD groups. The whole SIVD group had a higher proportion of hypertension, diabetes, hyperlipidemia, and drinking history; as well as higher levels of Hcy, Aβ40, and Aβ40/42 ratio; lower levels of Aβ42; more lacunes; and a larger volume of WMH. CBF in the whole brain and GM were lower than that in the HC group. Furthermore, SIVD patients showed poorer performance in MMSE, z-global, and all cognitive domains (including information processing, executive, attention, memory, language, and visuospatial). There were no significant differences in sex distribution, age, and years of education between the three groups (HC, SIVD-NCI, and SIVD-MCI). Compared with the HC group, patients in the SIVD-NCI group had higher rates of hypertension, hyperlipidemia, and drinking history; a larger volume of WMH; more lacunes; lower CBF in the whole brain and GM, and no significant difference in cognitive function between the two groups. Compared with the HC group, patients in the SIVD-MCI group had a higher proportion of hypertension, diabetes, hyperlipidemia, and drinking history; as well as higher levels of Hcy; more lacunes; and larger volume of WMH; lower CBF in the whole brain and GM. In addition, SIVD-MCI patients showed poorer performance in MMSE, z-global, and all cognitive domains than that in HCs. Compared with the SIVD-NCI group, patients in the SIVD-MCI group had more lacunes; a larger volume of WMH; and showed poorer performance in MMSE, z-global, and all cognitive domains. Differences in Aβ40, Aβ42, and Aβ40/42 ratio among the three groups of HC, SIVD-NCI, and SIVD-MCI were shown in Figure 1. Compared with the HC group, Aβ40 and Aβ40/42 ratios were significantly increased in SIVD-MCI group, while Aβ42 was significantly decreased (Figures 1A–C). There was no significant difference between SIVD-NCI and HC groups. Compared with the SIVD-NCI group, Aβ40 and Aβ40/42 ratios were significantly increased in the SIVD-MCI group (Figures 1A,C), with no significant difference in Aβ42 between the two groups.

**TABLE 1:** Demographic, clinical, and neuroimaging manifestations data.

Characteristics	HC (*n* = 74)	Whole SIVD (*n* = 74)	SIVD-NCI (*n* = 38)	SIVD-MCI (*n* = 36)	*P*
**Demographics**					
Age (years)	60.75 (8.09)	62.46 (7.81)^b^	61.95 (4.52)	63.14 (5.52)	0.119 ^d^
Male, *n* (%)	30 (40.5)	39 (52.7)^a^	23 (60.5)	19 (52.8)	0.114 ^a^
Education (years)	8.97 (2.87)	8.73 (2.55)^b^	9.13 (3.05)	8.25 (2.62)	0.355 ^d^
**Vascular risk factors**					
Hypertension, *n* (%)	10 (13.5)	37 (50.0)^a#^	16 (42.1)^#^	21 (58.3)^#^	**<0.001** ^a^
Diabetes, *n* (%)	8 (10.8)	20 (27.0)^a#^	9 (23.7)	11 (30.6)^#^	**0.032** ^a^
Hyperlipidemia, *n* (%)	10 (12.2)	27 (36.5)^a#^	13 (34.2)^#^	14 (38.9)^#^	**0.005** ^a^
Smoking history, *n* (%)	9 (10.0)	18 (24.3)^a^	9 (23.7)	9 (25.0)	0.158^a^
Drinking history, *n* (%)	9 (12.3)	28 (37.8)^a#^	13 (34.2)^#^	15 (41.6)^#^	**0.004** ^a^
**Laboratory examination**					
Glu (mmol/L)	5.40 (1.18)	5.63 (1.03)^b^	5.48 (0.88)	5.80 (1.15)	0.201^d^
TG (mmol/L)	1.45 (0.80)	1.48 (0.94)^b^	1.47 (0.98)	1.49 (0.91)	0.977^d^
TCH (mmol/L)	4.26 (0.76)	4.45 (1.25)^b^	4.31 (1.32)	4.59 (1.17)	0.267^d^
LDL-C (mmol/L)	3.02 (0.71)	3.23 (1.16)^b^	3.04 (1.23)	3.42 (1.05)	0.107^d^
HDL-C (mmol/L)	1.46 (0.38)	1.32 (0.32)^b^	1.33 (0.33)	1.31 (0.32)	0.068^d^
Hcy (μmol/L)	16.57 (5.06)	19.43 (10.11)^b#^	17.65 (6.90)	21.31 (12.49)^#^	**0.014** ^d^
hs-CRP (mg/L)	1.00 (1.25)	1.00 (1.32)^b^	1.00 (1.50)	1.05 (1.25)	0.666^d^
Aβ40 (pg/mL)	400.14 (32.42)	414.39 (34.49)^b#^	403.25 (35.59)	426.18 (29.42)^#†^	**<0.001** ^d^
Aβ42 (pg/mL)	66.44 (9.39)	62.76 (9.64)^b#^	63.94 (9.56)	61.52 (9.69)^#^	**0.037** ^d^
Aβ40/42 ratio	6.15 (1.06)	6.75 (1.15)^b#^	6.42 (1.01)	6.88 (1.22)^#†^	**<0.001** ^d^
APOE4, *n* (%)	11 (14.9)	19 (25.7)^a^	9 (23.7)	10 (27.8)	0.234^a^
**Neuropsychological tests**					
MMSE	27.89 (2.33)	25.85 (2.37)^b#^	26.97 (1.51)	24.64 (1.82)^#†^	**<0.001** ^d^
Information processing *z*-score	0.0 (0.77)	–0.85 (1.06)^b#^	–0.34 (0.78)	–1.30 (0.81)^#†^	**<0.001** ^d^
Executive *z*-score	0.0 (0.74)	–0.86 (1.21)^b#^	–0.39 (1.01)	–1.37 (1.21)^#†^	**<0.001** ^d^
Attention *z*-score	0.0 (0.89)	–0.54 (0.78)^b#^	–0.12 (0.69)	–0.99 (0.60)^#†^	**<0.001** ^d^
Memory *z*-score	0.0 (0.86)	–0.63 (1.04)^b#^	–0.21 (0.90)	–1.09 (1.01)^#†^	**<0.001** ^d^
Language *z*-score	0.0 (0.82)	–0.51 (0.90)^b#^	–0.15 (0.85)	–0.87 (0.82)^#†^	**<0.001** ^d^
Visuospatial *z*-score	0.0 (1.00)	–0.93 (1.49)^b#^	–0.33 (1.39)	–1.56 (1.34)^#†^	**<0.001** ^d^
Global *z*-score	0.0 (0.59)	–0.72 (0.79)^b#^	–0.24 (0.60)	–1.14 (0.66)^#†^	**<0.001** ^d^
**Neuroimaging**					
GMV (mL)	581.05 (45.34)	572.88 (51.47)^b^	583.800 (37.52)	561.36 (61.40)	0.081^d^
WMV (mL)	470.15 (55.26)	466.83 (42.35)^b^	476.72 (42.46)	456.39 (40.21)	0.189^d^
WMHV (ml)	1.06 (0.85)	7.93 (12.81)^c#^	7.11 (11.88)^#^	12.07 (13.94)^#†^	**<0.001** ^e^
Lacunes	0 (0)	1 (2)^c#^	1 (1)^#^	2 (3)^#†^	**<0.001** ^e^
CBF-Whole brain	0.9541 (0.0030)	0.9533 (0.0029)^b#^	0.9527 (0.0023)^#^	0.9524 (0.0026)^#^	**0.005** ^d^
CBF-GM	1.0053 (0.0126)	1.0007 (0.0122)^b#^	0.9985 (0.0149)^#^	0.9962 (0.0137)^#^	**0.002** ^d^
CBF-WM	0.9015 (0.0197)	0.8996 (0.0286)^b^	0.8963 (0.0209)	0.8947 (0.0272)	0.258^d^

Continuous variables with normal distribution (age, Glu, TG, TCH, LDL-C, HDL-C, Hcy, hs-CRP, Aβ40, Aβ42, Aβ40/42 ratio, Neuropsychological tests, GMV, WMV, CBF-Whole brain, CBF-GM, CBF-WM) were described by mean and standard deviation. Continuous variables with non-normal distribution (WMHV, Lacunes) were described by median and interquartile range. Categorical variables (sex, hypertension diabetes, hyperlipidemia, smoking history, drinking history, APOE4) were described by frequencies and percentages. *P*: The *p*-values of difference between HC, SIVD-NCI and SIVD-MCI. ^#^Whole SIVD, SIVD-NCI, SIVD-MCI group vs. HC group significantly different (*P* < 0.05). ^†^SIVD-MCI group vs. SIVD-NCI group significantly different (*P* < 0.05). a: Chi-square test. b: Two independent-samples *t*-test. c: Mann–Whitney *U* test. d: One-way analysis of variance (ANOVA) with a *post hoc* Bonferroni test. e: Kruskal–Wallis test. HC, healthy control; SIVD, subcortical ischemic vascular disease; NCI, no cognitive impairment; MCI, mild cognitive impairment; Glu, glucose; TCH, total cholesterol; TG, triglycerides; HDL-C, high density lipoprotein-cholesterol; LDL-C, low density lipoprotein-cholesterol; Hcy, homocysteine; hs-CRP, hypersensitive-C reactive protein; GMV, gray matter volume; WMV, white matter volume; WMHV, white matter hyperintensity volume; MMSE, Mini-Mental State Examination; GM, gray matter; WM, white matter; CBF, cerebral blood flow. Significant differences are indicated in bold.

### Receiver operating characteristic analysis of the serum Aβ40, Aβ42, Aβ40/42 levels in the diagnosis of the subcortical ischemic vascular disease and cognitive impairment

**FIGURE 2:**
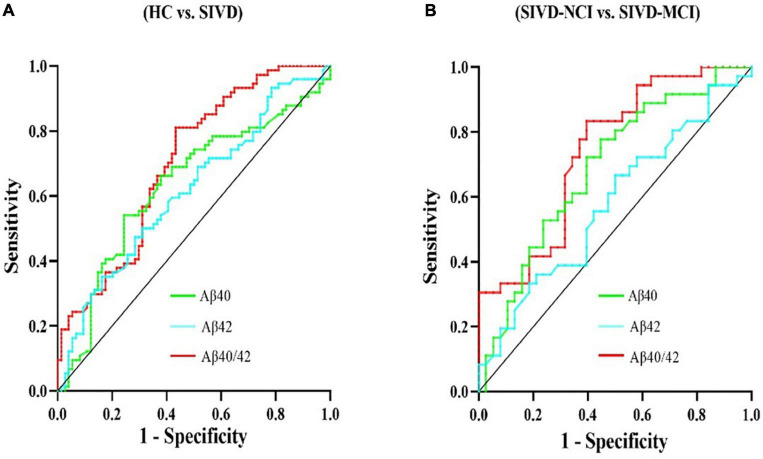
ROC analysis of the serum Aβ40, Aβ42, and Aβ40/42 ratio levels in the diagnosis of the SIVD and cognitive impairment. **(A)** ROC analyses of serum Aβ40, Aβ42, and Aβ40/42 levels for SIVD patients versus HC. **(B)** ROC analyses of serum Aβ40, Aβ42, and Aβ40/42 levels for SIVD-NCI versus SIVD-MCI. ROC, receiver operating characteristic; HC, healthy control; SIVD, subcortical ischemic vascular disease; NCI, no cognitive impairment; MCI, mild cognitive impairment; vs., versus.

Considering the above findings, we want to know more about which of the Aβ40, Aβ42, and Aβ40/42 has the greatest diagnostic value for SIVD and related cognitive impairments, we further determined the diagnostic value of serum Aβ40, Aβ42, and Aβ40/42 levels by constructing the ROC curve. As shown in Figure 2A, the AUCs for Aβ40 and Aβ42 to discriminate between HC and SIVD were 0.632 (*P* < 0.01, 95%CI: 0.541–0.723) and 0.614 (*P* < 0.05, 95%CI: 0.524–0.705), respectively; however, the AUC of Aβ40/42 ratio was increased to 0.702 (*P* < 0.001, 95%CI: 0.618–0.785). Similarly, The AUCs for Aβ40, Aβ42, and Aβ40/42 to discriminate between SIVD-NCI and SIVD-MCI were 0.689 (*P* < 0.01, 95%CI: 0.567–0.809), 0.578 (*P* = 0.245, 95%CI: 0.447–0.709), 0.741 (*P* < 0.001, 95%CI: 0.629–0.852), respectively (Figure 2B).

### Binary logistic regression analysis of the risk factors for cognitive impairment

To further examine the risk factors for cognitive impairment in SIVD patients, we took the occurrence of SIVD-MCI in SIVD patients as the dependent variable and included the influencing factors of *P* < 0.2 in the univariate logistic regression analysis as the independent variable. Finally, age, education, LDL-C, Hcy, Aβ40/Aβ42, WMHV, Lacunes were incorporated into the logistic regression model (Table 2). The result showed that Hcy {odds ratio [OR] = 1.152; 95% Confidence interval (CI = 1.004–1.323); *P* = 0.044}, WMHV (OR = 1.075; 95%CI = 1.017–1.137; *P* = 0.010) and Aβ40/42 ratio (OR = 2.237; 95%CI = 1.319–3.795; *P* = 0.003) were independent risk factors for SIVD-MCI after adjusting for confounders such as vascular risk factors and imaging markers of small vessel disease. Given the diagnostic value of the Aβ40/42 ratio and its impact on cognitive impairment in SIVD, we employed this indicator for further research in subsequent analysis.

**TABLE 2:** The results of the binary logistic regression analysis of clinical risk factors for cognitive impairment in SIVD-MCI patients.

	*B*	*SE*	Walds	*P*	OR (95%CI)
Age	0.048	0.062	0.598	0.439	1.049 (0.929–1.184)
Education	–0.014	0.108	0.017	0.897	0.986 (0.798–1.218)
LDL-C	0.439	0.265	2.745	0.098	1.551 (0.923–2.605)
Hcy	**0.142**	**0.070**	**4.048**	**0.044**	**1.152 (1.004**–**1.323)**
Aβ40/Aβ42	**0.805**	**0.270**	**8.921**	**0.003**	**2.237 (1.319**–**3.795)**
WMHV	**0.073**	**0.028**	**6.621**	**0.010**	**1.075 (1.017**–**1.137)**
Lacunes	0.065	0.180	0.129	0.719	1.067 (0.750–1.517)

LDL-C, low density lipoprotein-cholesterol; Hcy, homocysteine; WMHV, white matter hyperintensity volume; B, regression coefficient; SE, standard error; Walds, wald test; OR, odds ratio; CI, confidence interval. Significant differences are indicated in bold.

### Association between Aβ40/42 ratio, white matter hyperintensities volume, and cerebral blood flow

**FIGURE 3:**
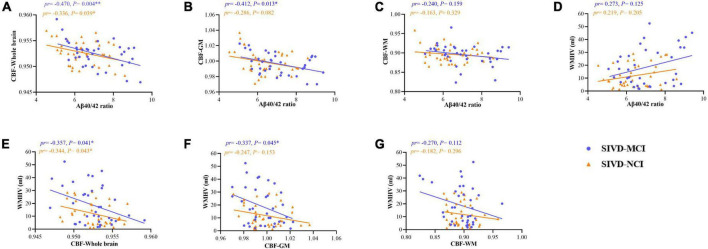
Scatter plots of correlation between serum Aβ40/42 ratio, WMHV, and CBF (including whole brain, GM, and WM) in patients with SIVD-MCI and SIVD-NCI. The blue dots represented the SIVD-MCI group, and the orange-yellow triangle represented the SIVD-NCI group. **(A)** Negative correlations between serum Aβ40/42 ratio and CBF in the whole brain. **(B)** Negative correlations between serum Aβ40/42 ratio and CBF in the GM. **(C)** Negative correlations between serum Aβ40/42 ratio and CBF in the WM. **(D)** Positive correlations between serum Aβ40/42 ratio and WMHV. **(E)** Negative correlations between CBF in the whole brain and WMHV. **(F)** Negative correlations between CBF in the GM and WMHV. **(G)** Negative correlations between CBF in the WM and WMHV. pr, partial correlation coefficient; GM, gray matter; WM, white matter; CBF, cerebral blood flow; WMHV, white matter hyperintensity volume. *P < 0.05, ^**^P < 0.01, respectively. (controlling for age, gender, education, vascular risk factors).

We performed a partial correlation analysis (after adjustment for age, sex, education, and vascular risk factors) on Aβ40/42 ratio with CBF values, and WMHV in patients with SIVD-NCI, SIVD-MCI, and whole SIVD. As shown in Figure 3, in the SIVD-MCI group, there were significant negative correlations between Aβ40/42 ratio and CBF in the whole brain (*pr* = –0.470, *P* = 0.004) (Figure 3A) and GM (*pr* = –0.412, *P* = 0.013) (Figure 3B). However, there was no significant correlation between Aβ40/42 ratio and CBF in WM (*pr* = –0.240, *P* = 0.159) (Figure 3C). In addition, WMHV was significantly negatively correlated with CBF in the whole brain (*pr* = –0.357, *P* = 0.041) (Figure 3E) and GM (*pr* = –0.337, *P* = 0.045) (Figure 3F), respectively. But there was no significant correlation between WMHV and CBF in WM (*pr* = –0.270, *P* = 0.112) (Figure 3G).

Similarly, we found that there were significant negative correlations between Aβ40/42 ratio and CBF in the whole brain (*pr* = –0.336, *P* = 0.039) (Figure 3A) in patients with SIVD-NCI, but the correlation between GM and WM was not significant (Figures 3B,C). At the same time, WMHV was significantly negatively correlated with CBF in the whole brain (*pr* = –0.344, *P* = 0.043) (Figure 3E), but the correlation with GM and WM was not significant (Figures 3F,G). As illustrated in Figure 3D, the Aβ40/42 ratio and WMHV were not significantly correlated in both SIVD-MCI and SIVD-NCI groups.

Correlations between Aβ40/42 ratio, WMHV, and CBF across the whole SIVD patients were shown in Supplementary Materials. As shown in Supplementary Figure 1, there were significant negative correlations between Aβ40/42 ratio and CBF in the whole brain (*pr* = –0.396, *P* < 0.001) (Supplementary Figure 1A) and GM (*pr* = –0.348, *P* = 0.003) (Supplementary Figure 1B). In addition, WMHV was significantly negatively correlated with CBF in the whole brain (*pr* = –0.349, *P* = 0.003) (Supplementary Figure 1E) and GM (*pr* = –0.290, *P* = 0.014) (Supplementary Figure 1F), respectively. Notably, the Aβ40/42 ratio was significantly positively correlated with WMHV (*pr* = 0.354, *P* = 0.002) (Supplementary Figure 1D).

### Group comparison of the cerebral blood flow

**FIGURE 4:**
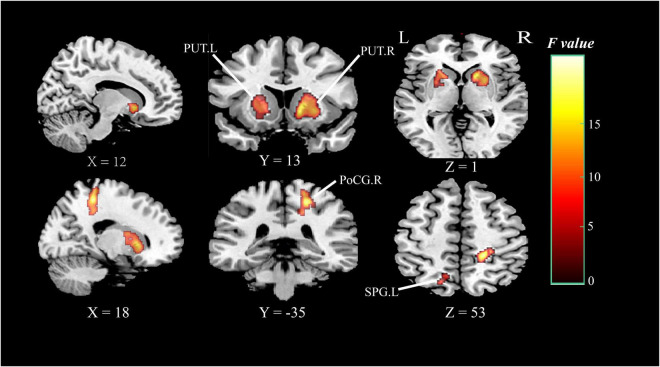
Brain regions of group differences in CBF values controlling for age gender and education (clusterlevel *P* < 0.05, FDR corrected, cluster-forming threshold at voxel-level *P* < 0.001); x, y, z, coordinates of primary peak locations in MNI space; the color bar represents the *F* values. PoCG, postcentral gyrus; SPG, superior parietal gyrus; PUT, putamen; L, left; R, right.

Analysis based on CBF data, Brain regions with significantly altered CBF controlled for age, gender, and education (*P* < 0.05, cluster-level FDR corrected, the cluster-forming threshold at voxel-level *P* < 0.001) were found in the right postcentral gyrus (PoCG.R), left superior parietal gyrus (SPG.L), right putamen (PUT.R), and left putamen (PUT.L) (Figure 4 and Table 3). Table 3 illustrated the post hoc results of the altered CBF between each pair of the groups. The CBF in the PoCG.R and SPG.L were significantly lower in the SIVD-NCI and SIVD-MCI groups than those of the HCs. Notably, the CBF values of PUT.R and PUT.L in the SIVD-NCI and SIVD-MCI groups were significantly higher than those of the HCs. However, there was no significant difference in CBF values between the SIVD-NCI and SIVD-MCI groups. We defined these regions with differing perfusion as the regions of interest (ROIs) for further correlation analyses.

**TABLE 3:** Brain regions with significant differences in CBF between patients with HCs, SIVD-NCI, and SIVD-MCI.

Brain regions (AAL)	Cluster size (voxels)	MNI coordinates			*F* value	Peak *T* value		
		*x*	*y*	*z*		SIVD-NCI vs. HCs	SIVD-MCI vs. HCs	SIVD-MCI vs. SIVD-NCI
**Decreased regions**								
PoCG.R	262	20	–36	56	19.31	5.48***	6.01***	0.39
SPG.L	144	–16	–64	42	10.82	3.56***	4.32***	0.33
**Increased regions**								
PUT.R	670	14	16	–4	17.08	–4.06***	–5.86***	–0.33
PUT.L	348	–18	4	14	11.55	–3.35**	–4.80***	–0.67

MNI, Montreal Neurological Institute; x, y, z, coordinates of primary peak locations in MNI space. CBF, cerebral blood flow; HCs, healthy controls, SIVD, subcortical ischemic vascular disease; NCI, no cognitive impairment; MCI, mild cognitive impairment; PoCG, postcentral gyrus; SPG, superior parietal gyrus; PUT, putamen; L, left; R, right. *P* < 0.05, cluster-level FDR corrected, cluster-forming threshold at voxel-level *P* < 0.001. **P < 0.01; ***P < 0.001.

### Association between cerebral blood flow, white matter hyperintensities volume and neuropsychological tests

**FIGURE 5:**
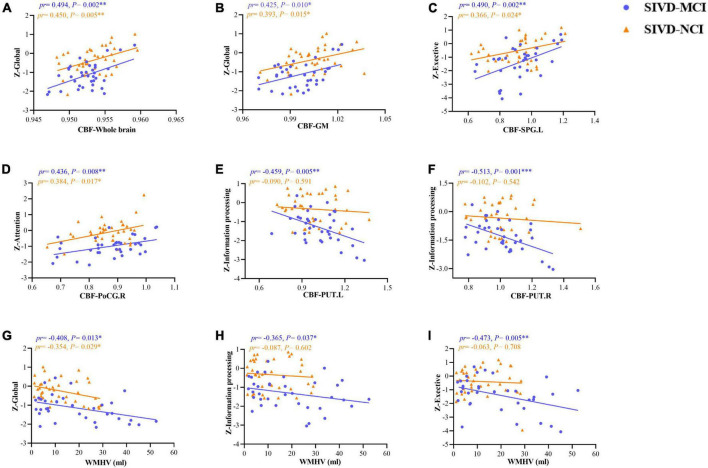
Scatter plots of correlation between CBF, WMHV, and neuropsychological tests in patients with SIVD-MCI and SIVD-MCI. The blue dots represented the SIVD-MCI group, and the orange-yellow triangle represented the SIVD-NCI group. **(A)** Positive correlations between CBF in the whole brain and *z*-scores in global. **(B)** Positive correlations between CBF in the GM and *z*-scores in global. **(C)** Positive correlations between CBF in SPG.L and *z*-scores in exective function. **(D)** Positive correlations between CBF in PoCG.R and z-scores in attention function. **(E)** Negative correlations between CBF in PUT.L and *z*-scores in information processing function. **(F)** Negative correlations between CBF in PUT.R and *z*-scores in information processing function. **(G)** Negative correlations between WMHV and *z*-scores in global cognition. **(H)** Negative correlations between WMHV and *z*-scores in information processing function. **(I)** Negative correlations between WMHV and *z*-scores in executive function. pr, partial correlation coefficient; CBF, cerebral blood flow; GM, gray matter; PoCG, postcentral gyrus; SPG, superior parietal gyrus; PUT, putamen; WMHV, white matter hyperintensity volume; L, left; R, right. *P < 0.05; ^**^P < 0.01; ^***^P < 0.001. (Controlling for age, gender, education, vascular risk factors).

Figure 5 illustrated the associations between CBF values in the whole brain, GM, ROIs, WMHV, and neuropsychological tests in patients with SIVD-MCI and SIVD-NCI. In the SIVD-MCI group, the CBF of the whole brain (*pr* = 0.494, *P* = 0.002) (Figure 5A) and GM (*pr* = 0.425, *P* = 0.010) (Figure 5B) was positively correlated with global cognitive function. The CBF of SPG.L was positively correlated with executive function (*pr* = 0.490, *P* = 0.002) (Figure 5C). Moreover, CBF of PoCG.R was positively correlated with attention function (*pr* = 0.436, *P* = 0.008) (Figure 5D). Interestingly, CBF of PUT.L (*pr* = –0.459, *P* = 0.005) and PUT.R (*pr* = –0.513, *P* = 0.001) were negatively correlated with information processing speed (Figures 5E,F). It is worth noting that WMHV was significantly negatively correlated with global cognitive function (*pr* = –0.408, *P* = 0.013) (Figure 5G), information processing speed (*pr* = –0.365, *P* = 0.037) (Figure 5H) and executive function (*pr* = –0.473, *P* = 0.005) (Figure 5I).

**FIGURE 6:**
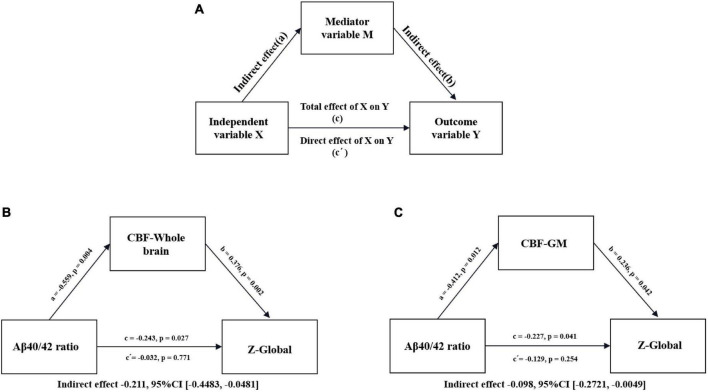
Conceptual diagram of mediation analysis between Aβ40/42 ratio, CBF, and global cognition in patients with SIVD-MCI. **(A)** Conceptual diagram of a mediation analysis model with one mediator. Total effect of X on Y (c) = indirect effect of X on Y through M (a × b) + direct effect of X on Y (c’). **(B)** Serum Aβ40/42 ratio (X), CBF in the whole brain (M), *z*-score of global (Y). **(C)** Serum Aβ40/42 ratio (X), CBF in the GM (M), *z*-score of global (Y). Path coefficients with *P*-values. GM, gray matter; CBF, cerebral blood flow; CI, confidence interval.

Similarly, we found that there were significant positive correlations between global cognitive function and CBF in the whole brain (*pr* = 0.450, *P* = 0.005) (Figure 5A) and GM (*pr* = 0.393, *P* = 0.015) (Figure 5B) in patients with SIVD-NCI. The CBF of SPG.L was positively correlated with executive function (*pr* = 0.366, *P* = 0.024) (Figure 5C). Moreover, CBF of PoCG.R was also positively correlated with attention function (*pr* = 0.384, *P* = 0.017) (Figure 5D). Unfortunately, there was no significant correlation between the CBF value of the bilateral PUT and information processing speed (Figures 5E,F). WMHV was significantly negatively correlated with global cognition (*pr* = 0.354, *P* = 0.029) (Figure 5G), but not with information processing speed and executive function (Figures 5H,I).

Correlations between CBF, WMHV, CBF, and neuropsychological tests in the whole SIVD patients were shown in Supplementary Materials. As shown in Supplementary Figure 2, the CBF of the whole brain (*pr* = 0.485, *P* < 0.001) (Supplementary Figure 2A) and GM (*pr* = 0.462, *P* < 0.001) (Supplementary Figure 2B) was positively correlated with global cognition. The CBF of SPG.L and PoCG.R were positively correlated with executive function (*pr* = 0.360, *P* = 0.002) (Supplementary Figure 2C) and attention function (*pr* = 0.340, *P* = 0.004) (Supplementary Figure 2D), respectively. Moreover, CBF of PUT.L (*pr* = –0.408, *P* < 0.001) and PUT.R (*pr* = –0.259, *P* = 0.029) were negatively correlated with information processing speed (Supplementary Figure 2E,F). In addition, WMHV was significantly negatively correlated with global cognition (*pr* = –0.459, *P* < 0.001) (Supplementary Figure 2G), information processing speed (*pr* = –0.342, *P* = 0.003) (Supplementary Figure 2H) and executive function (*pr* = –0.358, *P* = 0.002) (Supplementary Figure 2I).

### Mediation analysis

To examine the relationship between the CBF (whole brain and GM), Aβ40/42 ratio, and cognitive function, we performed a mediation analysis to identify whether alterations in serum Aβ40/42 ratio could cause CBF changes that affect cognitive dysfunction. As shown in Figure 6, the relationship between Aβ40/42 ratio and global z-scores was significantly mediated by the CBF of the whole brain (indirect effect = –0.211, 95%CI [–0.4483, –0.0481]) in patients with SIVD-MCI (Figure 6B). We also found that the CBF of the GM significantly mediated the relationship between Aβ40/42 ratio and global *z*-scores (indirect effect = –0.098, 95%CI [–0.2721, –0.0049]) in SIVD-MCI patients (Figure 6C). Regrettably, we did not find a similar mediating effect in SIVD-NCI patients. At the same time, we performed a mediation analysis on all whole SIVD patients. As shown in Supplementary Figure 3, the relationship between Aβ40/42 ratio and global *z*-scores was significantly mediated by the CBF of the whole brain (indirect effect = –0.092, 95%CI [–0.2206, –0.0124]) (Supplementary Figure 3A). Similarly, the CBF of the GM significantly mediated the relationship between Aβ40/42 ratio and global z-scores (indirect effect = –0.137, 95%CI [–0.2528, –0.0610]) (Supplementary Figure 3B).

### Stratified analysis

The age range of the subjects in our study was 50–80 years, and the larger age range inevitably had an impact on cognition and other age-related findings. Therefore, we performed a stratified analysis according to age to test the robustness of our results. Due to the small sample size of our case group, we did not further stratify the two subgroups of SIVD patients by age, finally, we divided the HC and SIVD groups into 50–65 and 66–80-years old groups by age. We performed matched comparisons between the two groups and presented the results in the Supplementary material (Supplementary Table 1 and Supplementary Figure 4–7). We found that the results stratified by age were consistent with our previous results, which also verified the stability of our results.

## Discussion

We aimed to investigate the altered serum Aβ and cerebral perfusion and their associations with cognitive function in patients with SIVD. Consistent with our hypothesis, the serum Aβ40 and Aβ40/42 ratio levels of SIVD-MCI were significantly higher than those of the SIVD-NCI and HCs, and serum Aβ42 levels of SIVD-MCI were lower than those of the HCs. Serum Aβ40/42 ratio could not only effectively identify SIVD and its related cognitive impairment, but also was an independent risk factor for cognitive impairment. Patients with SIVD-MCI showed decreased global cerebral perfusion (including whole brain and GM), which was negatively associated with a higher Aβ40/42 ratio, and positively correlated with global z-scores. Similarly, CBF in the whole brain was negatively associated with a higher Aβ40/42 ratio in SIVD-NCI patients. SIVD-NCI and SIVD-MCI patients manifested with reduced regional cerebral perfusion in PoCG.R and SPG.L, and compensatory elevation of regional cerebral perfusion in bilateral putamen. The decreased CBF values of PoCG.R and SPG.L were positively correlated with executive and attention function, respectively. In contrast, the increased CBF value of bilateral putamen was negatively correlated with the speed of information processing in patients with SIVD-MCI. In addition, WMHV was positively correlated with Aβ40/42 ratio and negatively correlated with CBF in the whole brain and GM. Mediation analysis suggested that the relationship between the Aβ40/42 ratio and cognitive impairment was partially mediated by the altered CBF.

A growing body of evidence showed that abnormal blood Aβ levels appear in the early stages of cognitive impairment (Devanand et al., 2011; Chen et al., 2021). Aβ deposition in brain tissue is one of the main causes of cognitive impairment (Ross and Poirier, 2004), and most Aβ produced in the brain is degraded by the ubiquitin-proteasome system and autophagy system (Tarasoff-Conway et al., 2015; Xin et al., 2018), or cleared by proteases secreted by astrocytes and microglia (McDonald et al., 2016). While the remaining Aβ can enter the peripheral blood circulation through the blood-cerebrospinal fluid barrier, blood-brain barrier, perivascular drainage, arachnoid villi, lymphatic drainage, etc. (Xin et al., 2018), which may be the reason for the increase of Aβ levels in peripheral blood, thus the changes in peripheral blood Aβ levels also reflect the progress of the disease.

Our results were consistent with previous findings by Kim et al., who also found that an increased Aβ40/Aβ42 ratio is more valuable in distinguishing AD from controls (Kim et al., 2015). Similarly, in this study, we also took the Aβ40/Aβ42 ratio for further study and believed that it better reflects the changes in Aβ than Aβ40 or Aβ42 alone, in addition, Aβ40/Aβ42 ratio was an independent risk factor for cognitive impairment in SIVD patients. However, there are also some studies showing that in MCI patients, plasma Aβ42 concentrations are only modestly reduced, while Aβ40 levels remain unchanged (Janelidze et al., 2016). Previous studies have shown that when suffering cerebral hypoperfusion or reduced cerebral blood flow, the overproduction and secretion of Aβ into the circulation increases peripheral levels (Bennett et al., 2000). Compared with shorter Aβ40, Aβ42 is more likely to accumulate in brain amyloid plaques and not be cleared easily (Hardy and Selkoe, 2002), which may be the reason for the increase of serum Aβ40 and the relatively low level of Aβ42.

Based on cerebral blood flow values acquired by ASL, firstly, at the level of whole-brain perfusion, we found that CBF values in the whole brain and GM were decreased in SIVD-NCI and SIVD-MCI patients and correlated with overall cognitive decline. This indicates that the decline of global cerebral perfusion is an important influencing factor of cognitive impairment in SIVD patients, which is consistent with the results of most previous studies (Mokhber et al., 2021). Furthermore, in specific regions, we found that SIVD-NCI and SIVD-MCI patients exhibited reduced CBF in the SPG.L and PoCG.R regions relative to HCs, which is consistent with the findings of some previous studies (Liu et al., 2021), but others showed different results (Sun et al., 2016). Notably, our results showed the presence of both hypoperfused and hyperperfused regions, showing hyperperfusion in the bilateral putamen. This may be attributed to compensatory mechanisms in the early pathological process of the disease to maintain normal cognitive function (van der Thiel et al., 2019).

Stable CBF is fundamental to maintaining brain cognitive function, we performed voxel analysis to explore the relationship between regional CBF changes and cognitive deficits. Decreased CBF in the SPG.L and PoCG.R regions was associated with worse executive and attentional function, respectively. Previous studies have shown that the parietal cortex is a key region of the executive control network and is closely related to inhibitory control, attention, working memory, planning, and response (Uddin et al., 2011). The attentional network, also known as the fronto-parietal or executive attention network, is mainly composed of the dorsolateral prefrontal, posterior parietal, and lateral temporal lobes (Markett et al., 2014). Thus, hypoperfusion in the parietal lobe, including the SPG.L and PoCG.R regions, may lead to disruption of subcortical circuits in the parietal lobe, and disruption of interactions with the frontal lobe leading to impaired executive or attentional function. In addition, we found that in SIVD-MCI and the whole SIVD patients, increased bilateral putamen perfusion was negatively associated with information processing speed. The basal ganglia area, including the putamen, is a key component of the cortical-basal ganglia-thalamic circuit (Haber and Calzavara, 2009). While the normal completion of information processing depends on the structural and functional integrity of the cortex, basal ganglia, and thalamus (Leisman and Melillo, 2013), the increased perfusion in the subcortical putamen may be a compensation for the damage to the cortical-basal ganglia-thalamic circuit to maintain normal operation of information processing functions.

A mass of research has been done on the relationship between Aβ and CBF, but the exact causal relationship between them has not yet been clarified. However, in recent years, studies have found that amyloid-β load is associated with reduced cerebral blood flow in the control group, while brain atrophy is predominant in late mild cognitive impairment and dementia, indicating that the mechanism of CBF reduction occurs in the early stage of the disease, which may be an initiating factor that activates the amyloid cascade (Mattsson et al., 2014; Wierenga et al., 2014). There are also some studies from the biological point of view of Aβ itself and found that Aβ can activate microglia and astrocytes to produce inflammatory factors such as interleukin-1β (Kamphuis et al., 2015), and these inflammatory cytokines have been confirmed to decrease CBF by releasing endothelin (Murray et al., 2014). In addition, Aβ-induced reactive oxygen species directly act on cerebral arteries to promote vasoconstriction and reduce CBF (Niwa et al., 2001). Our results show that the serum Aβ40/40 ratio is negatively correlated with the CBF of the whole brain, and we use the serum Aβ level to reflect the level of Aβ in the brain tissue, which suggests that Aβ may increase with the decrease of perfusion, but from another point of view, it can also be considered that the CBF gradually decreases with an increase of Aβ level. Therefore, in our cross-sectional study, the causal relationship between the CBF and Aβ cannot be obtained yet, and a large number of longitudinal follow-up studies are needed to confirm in the future.

Exploring the mechanism of cerebral perfusion in disease will help us to further identify potential therapeutic options to delay or treat cognitive impairment closely related to cerebrovascular, including vascular dementia or AD. Applying mediation analysis, we examined the correlation between serum Aβ levels, CBF, and cognitive function in SIVD patients. Our results suggest that the relationship between serum Aβ levels and cognitive deficits in SIVD-MCI patients is mediated by changes in CBF, suggesting that altered CBF in SIVD patients is a key factor in cognitive impairment. However, regarding the relationship between serum Aβ levels and cognitive impairment, it may not be directly caused by changes in CBF. Recently, more and more studies have found that blood Aβ levels are closely related to markers of cerebral small vessel disease, especially white matter hyperintensity (WMH) and lacunes. Low circulating levels of Aβ40 and Aβ42 were associated with increased WMH progression in a follow-up study in a community-based population without dementia (Kaffashian et al., 2014). In another longitudinal study of RUN DMC up to 9 years, it was found that cross-sectionally, plasma Aβ40 levels were elevated in the severe WMH and lacunes groups, and longitudinally, plasma Aβ40 levels were significantly elevated in participants with lacunes and WMH progression groups (van Leijsen et al., 2018). In our study, we accurately calculated WMHV by T2 flair imaging, whereby we further explored the relationship between Aβ40/42 ratio and WMHV. Although no significant correlation was found between the SIVD-NCI and SIVD-MCI subgroups, which may be due to the small sample size. However, Aβ40/42 ratio was found to be positively correlated with WMHV in the whole SIVD patients. Recent studies found that Aβ0 and Aβ2 levels in cerebrospinal fluid were associated with WMHV (Osborn et al., 2018; Guo et al., 2021), suggesting that Aβ40 and Aβ42 levels may be associated with white matter damage in SIVD patients. At the same time, we also found that WMHV was negatively correlated with CBF in the whole brain and GM, overall cognition, information processing speed, and executive function, while Jann et al. (2021) also found that WMHV was associated with whole-brain perfusion and executive function in cerebral small vessel disease patients, which was similar to our findings. Therefore, we had reasons to speculate that Aβ may further cause cognitive decline by reducing CBF leading to WMH. In the future, more research is needed to explore this pathway to understand the possible mechanism of Aβ-induced cognitive impairment in SIVD patients.

There are still several limitations with our study that need to be addressed. First, our findings are limited to a small sample, which may affect the results of serum Aβ assays, thereby affecting the comprehensive interpretation of serum Aβ levels, CBF alteration, and cognitive impairment in SIVD patients. Secondly, the sample we tested is serum, it may affect by many peripheral factors. Future research needs to collect cerebrospinal fluid samples to exclude interfering factors as much as possible. Third, we did not exclude the effect of drugs on cognitive assessment, such as antihypertensive drugs, hypoglycemic drugs, etc., which may affect cognitive function. Fourth, we did not focus on the effects of other cerebral small vessel disease markers such as enlarged perivascular spaces and microbleeds on cognitive function. Lastly, we only performed a cross-sectional study of SIVD patients, subsequent studies should focus on follow-up studies of SIVD patients and observe the relationship between serum Aβ levels and dynamic changes in CBF, as well as the underlying mechanisms of cognitive impairment in SIVD patients.

## Conclusion

Altered serum Aβ levels in SIVD patients are associated with cognitive impairment, which may be mediated through alteration in CBF. In addition, serum Aβ levels were closely related to alterations in cerebral perfusion, and regional changes in cerebral perfusion were associated with cognitive deficits in specific domains. The serum Aβ40/42 ratio may be a potential biomarker that can complement current methods for the prediction and diagnosis of cognitive impairment in SIVD patients. In addition, serum Aβ may play a role in cognitive function by regulating CBF, which provides new insights into the intervention, treatment, and prevention of cognitive impairment in SIVD.

## Data availability statement

The original contributions presented in this study are included in the article/Supplementary material, further inquiries can be directed to the corresponding author.

## Ethics statement

The studies involving human participants were reviewed and approved by Ethics Committees of the First Affiliated Hospital of Anhui Medical University (Reference no. Quick-PJ2022-09-09). The patients/participants provided their written informed consent to participate in this study.

## Author contributions

WZ and ML acquired and analyzed the data, conceptualized the study, and drafted the manuscript for intellectual content. CH, CL, and JY contributed to the acquisition and analysis of data. WZ and CZ contributed to MRI scan and image data analysis. KW, XZ, XQZ, and ZS designed and conceptualized the study, obtained funding, and revised the manuscript for intellectual content. All authors contributed to the article and approved the submitted version.

## Footnotes

1
http://www.neuro.uni-jena.de/cat

2
www.statistical-modelling.de/lst.html

3
http://www.fil.ion.ucl.ac.uk/spm/

4
https://www.nitrc.org/projects/mricron

5
http://www.processmacro.org/

## References

[B1] Bell R. D. Zlokovic B. V. (2009). Neurovascular mechanisms and blood-brain barrier disorder in Alzheimer’s disease. *Acta Neuropathol.* 118 103–113. 10.1007/s00401-009-0522-523 19319544 PMC2853006

[B2] Bennett S. A. Pappas B. A. Stevens W. D. Davidson C. M. Fortin T. Chen J. (2000). Cleavage of amyloid precursor protein elicited by chronic cerebral hypoperfusion. *Neurobiol. Aging* 21 207–214. 10.1016/s0197-4580(00)00131-137 10867205

[B3] Bibl M. Esselmann H. Mollenhauer B. Weniger G. Welge V. Liess M. (2007). Blood-based neurochemical diagnosis of vascular dementia: a pilot study. *J. Neurochem.* 103 467–474. 10.1111/j.1471-4159.2007.04763.x 17662050

[B4] Buxton R. B. Frank L. R. Wong E. C. Siewert B. Warach S. Edelman R. R. (1998). A general kinetic model for quantitative perfusion imaging with arterial spin labeling. *Magn. Reson. Med.* 40 383–396. 10.1002/mrm.1910400308 9727941

[B5] Chen Y. R. Liang C. S. Chu H. Voss J. Kang X. L. O’Connell G. (2021). Diagnostic accuracy of blood biomarkers for Alzheimer’s disease and amnestic mild cognitive impairment: a meta-analysis. *Ageing Res. Rev.* 71:101446. 10.1016/j.arr.2021.101446 34391944

[B6] de la Torre J. C. Mussivand T. (1993). Can disturbed brain microcirculation cause Alzheimer’s disease? *Neurol. Res.* 15 146–153. 10.1080/01616412.1993.11740127 8103579

[B7] Devanand D. P. Schupf N. Stern Y. Parsey R. Pelton G. H. Mehta P. (2011). Plasma Aβ and PET PiB binding are inversely related in mild cognitive impairment. *Neurology* 77 125–131. 10.1212/WNL.0b013e318224afb7 21715709 PMC3140071

[B8] Galluzzi S. Sheu C. F. Zanetti O. Frisoni G. B. (2005). Distinctive clinical features of mild cognitive impairment with subcortical cerebrovascular disease. *Dement. Geriatr. Cogn. Disord.* 19 196–203. 10.1159/000083499 15677867

[B9] Gandy S. (2005). The role of cerebral amyloid beta accumulation in common forms of Alzheimer disease. *J. Clin. Invest.* 115 1121–1129. 10.1172/jci25100 15864339 PMC1087184

[B10] Gorelick P. B. Scuteri A. Black S. E. Decarli C. Greenberg S. M. Iadecola C. (2011). Vascular contributions to cognitive impairment and dementia: a statement for healthcare professionals from the american heart association/american stroke association. *Stroke* 42 2672–2713. 10.1161/STR.0b013e3182299496 21778438 PMC3778669

[B11] Guo T. Landau S. M. Jagust W. J. (2021). Age, vascular disease, and Alzheimer’s disease pathologies in amyloid negative elderly adults. *Alzheimers Res. Ther.* 13:174. 10.1186/s13195-021-00913-915 PMC8520216 34654465

[B12] Haber S. N. Calzavara R. (2009). The cortico-basal ganglia integrative network: the role of the thalamus. *Brain Res. Bull.* 78 69–74. 10.1016/j.brainresbull.2008.09.013 18950692 PMC4459637

[B13] Hardy J. Selkoe D. J. (2002). The amyloid hypothesis of Alzheimer’s disease: progress and problems on the road to therapeutics. *Science* 297 353–356. 10.1126/science.1072994 12130773

[B14] Inoue Y. Tanaka Y. Hata H. Hara T. (2014). Arterial spin-labeling evaluation of cerebrovascular reactivity to acetazolamide in healthy subjects. *AJNR Am. J. Neuroradiol.* 35 1111–1116. 10.3174/ajnr.A3815 24371025 PMC7965130

[B15] Janelidze S. Stomrud E. Palmqvist S. Zetterberg H. van Westen D. Jeromin A. (2016). Plasma β-amyloid in Alzheimer’s disease and vascular disease. *Sci. Rep.* 6:26801. 10.1038/srep26801 27241045 PMC4886210

[B16] Jann K. Shao X. Ma S. J. Cen S. Y. D’Orazio L. Barisano G. (2021). Evaluation of cerebral blood flow measured by 3D PCASL as biomarker of Vascular Cognitive Impairment and Dementia (VCID) in a cohort of elderly latinx subjects at risk of small vessel disease. *Front. Neurosci.* 15:627627. 10.3389/fnins.2021.627627 33584191 PMC7873482

[B17] Jokinen H. Kalska H. Mantyla R. Pohjasvaara T. Ylikoski R. Hietanen M. (2006). Cognitive profile of subcortical ischaemic vascular disease. *J. Neurol. Neurosurg. Psychiatry* 77 28–33. 10.1136/jnnp.2005.069120 16361588 PMC2117424

[B18] Kaffashian S. Tzourio C. Soumaré A. Dufouil C. Zhu Y. Crivello F. (2014). Plasma β-amyloid and MRI markers of cerebral small vessel disease: three-city Dijon study. *Neurology* 83 2038–2045. 10.1212/wnl.0000000000001038 25355827

[B19] Kamphuis W. Kooijman L. Orre M. Stassen O. Pekny M. Hol E. M. (2015). GFAP and vimentin deficiency alters gene expression in astrocytes and microglia in wild-type mice and changes the transcriptional response of reactive glia in mouse model for Alzheimer’s disease. *Glia* 63 1036–1056. 10.1002/glia.22800 25731615

[B20] Keage H. A. Carare R. O. Friedland R. P. Ince P. G. Love S. Nicoll J. A. (2009). Population studies of sporadic cerebral amyloid angiopathy and dementia: a systematic review. *BMC Neurol.* 9:3. 10.1186/1471-2377-9-3 19144113 PMC2647900

[B21] Kim H. J. Park K. W. Kim T. E. Im J. Y. Shin H. S. Kim S. (2015). Elevation of the Plasma Aβ40/Aβ42 ratio as a diagnostic marker of sporadic early-onset Alzheimer’s disease. *J. Alzheimers. Dis.* 48 1043–1050. 10.3233/jad-143018 26444752

[B22] Kisler K. Nelson A. R. Rege S. V. Ramanathan A. Wang Y. Ahuja A. (2017). Pericyte degeneration leads to neurovascular uncoupling and limits oxygen supply to brain. *Nat. Neurosci.* 20 406–416. 10.1038/nn.4489 28135240 PMC5323291

[B23] Korte N. Nortley R. Attwell D. (2020). Cerebral blood flow decrease as an early pathological mechanism in Alzheimer’s disease. *Acta Neuropathol.* 140 793–810. 10.1007/s00401-020-02215-w 32865691 PMC7666276

[B24] Lee J. H. Kim S. H. Kim G. H. Seo S. W. Park H. K. Oh S. J. (2011). Identification of pure subcortical vascular dementia using 11C-Pittsburgh compound B. *Neurology* 77 18–25. 10.1212/WNL.0b013e318221acee 21593437

[B25] Leisman G. Melillo R. (2013). The basal ganglia: motor and cognitive relationships in a clinical neurobehavioral context. *Rev. Neurosci.* 24 9–25. 10.1515/revneuro-2012-2067 23241666

[B26] Liu X. Cheng R. Chen L. Gong J. Luo T. Lv F. (2021). Altered neurovascular coupling in subcortical ischemic vascular disease. *Front. Aging Neurosci.* 13:598365. 10.3389/fnagi.2021.598365 34054499 PMC8149589

[B27] Markett S. Reuter M. Montag C. Voigt G. Lachmann B. Rudorf S. (2014). Assessing the function of the fronto-parietal attention network: insights from resting-state fMRI and the attentional network test. *Hum. Brain Mapp.* 35 1700–1709. 10.1002/hbm.22285 23670989 PMC6869384

[B28] Mattsson N. Tosun D. Insel P. S. Simonson A. Jack C. R. Jr. Beckett L. A. (2014). Association of brain amyloid-β with cerebral perfusion and structure in Alzheimer’s disease and mild cognitive impairment. *Brain* 137(Pt 5), 1550–1561. 10.1093/brain/awu043 24625697 PMC3999717

[B29] McDonald C. L. Hennessy E. Rubio-Araiz A. Keogh B. McCormack W. McGuirk P. (2016). Inhibiting TLR2 activation attenuates amyloid accumulation and glial activation in a mouse model of Alzheimer’s disease. *Brain Behav. Immun.* 58 191–200. 10.1016/j.bbi.2016.07.143 27422717

[B30] Mokhber N. Shariatzadeh A. Avan A. Saber H. Babaei G. S. Chaimowitz G. (2021). Cerebral blood flow changes during aging process and in cognitive disorders: a review. *Neuroradiol. J.* 34 300–307. 10.1177/19714009211002778 33749402 PMC8447819

[B31] Mueller K. Lepsien J. Möller H. E. Lohmann G. (2017). Commentary: cluster failure: why fMRI inferences for spatial extent have inflated false-positive rates. *Front. Hum. Neurosci.* 11:345. 10.3389/fnhum.2017.00345 28701944 PMC5487467

[B32] Murray K. N. Girard S. Holmes W. M. Parkes L. M. Williams S. R. Parry-Jones A. R. (2014). Systemic inflammation impairs tissue reperfusion through endothelin-dependent mechanisms in cerebral ischemia. *Stroke* 45 3412–3419. 10.1161/strokeaha.114.006613 25228257 PMC4363555

[B33] Niwa K. Porter V. A. Kazama K. Cornfield D. Carlson G. A. Iadecola C. (2001). A beta-peptides enhance vasoconstriction in cerebral circulation. *Am. J. Physiol. Heart Circ. Physiol.* 281 H2417–H2424. 10.1152/ajpheart.2001.281.6.H2417 11709407

[B34] Osborn K. E. Liu D. Samuels L. R. Moore E. E. Cambronero F. E. Acosta L. M. Y. (2018). Cerebrospinal fluid β-amyloid(42) and neurofilament light relate to white matter hyperintensities. *Neurobiol. Aging* 68 18–25. 10.1016/j.neurobiolaging.2018.03.028 29702372 PMC6085839

[B35] Roman G. C. Erkinjuntti T. Wallin A. Pantoni L. Chui H. C. (2002). Subcortical ischaemic vascular dementia. *Lancet Neurol.* 1 426–436. 10.1016/s1474-4422(02)00190-194 12849365

[B36] Ross C. A. Poirier M. A. (2004). Protein aggregation and neurodegenerative disease. *Nat. Med.* 10(Suppl.), S10–S17. 10.1038/nm1066 15272267

[B37] Sachdev P. Kalaria R. O’Brien J. Skoog I. Alladi S. Black S. E. (2014). Diagnostic criteria for vascular cognitive disorders: a VASCOG statement. *Alzheimer Dis. Assoc. Disord.* 28 206–218. 10.1097/WAD.0000000000000034 24632990 PMC4139434

[B38] Schuff N. Matsumoto S. Kmiecik J. Studholme C. Du A. Ezekiel F. (2009). Cerebral blood flow in ischemic vascular dementia and Alzheimer’s disease, measured by arterial spin-labeling magnetic resonance imaging. *Alzheimers Dement.* 5 454–462. 10.1016/j.jalz.2009.04.1233 19896584 PMC2802181

[B39] Snyder H. M. Corriveau R. A. Craft S. Faber J. E. Greenberg S. M. Knopman D. (2015). Vascular contributions to cognitive impairment and dementia including Alzheimer’s disease. *Alzheimers Dement.* 11 710–717. 10.1016/j.jalz.2014.10.008 25510382 PMC4731036

[B40] Sun Y. Cao W. Ding W. Wang Y. Han X. Zhou Y. (2016). Cerebral blood flow alterations as assessed by 3D ASL in cognitive impairment in patients with subcortical vascular cognitive impairment: a marker for disease severity. *Front. Aging Neurosci.* 8:211. 10.3389/fnagi.2016.00211 27630562 PMC5005930

[B41] Tarasoff-Conway J. M. Carare R. O. Osorio R. S. Glodzik L. Butler T. Fieremans E. (2015). Clearance systems in the brain-implications for Alzheimer disease. *Nat. Rev. Neurol.* 11 457–470. 10.1038/nrneurol.2015.119 26195256 PMC4694579

[B42] Uddin L. Q. Supekar K. S. Ryali S. Menon V. (2011). Dynamic reconfiguration of structural and functional connectivity across core neurocognitive brain networks with development. *J. Neurosci.* 31 18578–18589. 10.1523/jneurosci.4465-11.2011 22171056 PMC3641286

[B43] van der Thiel M. Rodriguez C. Van De Ville D. Giannakopoulos P. Haller S. (2019). Regional cerebral perfusion and cerebrovascular reactivity in elderly controls with subtle cognitive deficits. *Front. Aging Neurosci.* 11:19. 10.3389/fnagi.2019.00019 30837863 PMC6390712

[B44] van Leijsen E. M. C. Kuiperij H. B. Kersten I. Bergkamp M. I. van Uden I. W. M. Vanderstichele H. (2018). Plasma Aβ (Amyloid-β) levels and severity and progression of small vessel disease. *Stroke* 49 884–890. 10.1161/strokeaha.117.019810 29540613

[B45] Vilar-Bergua A. Riba-Llena I. Nafria C. Bustamante A. Llombart V. Delgado P. (2016). Blood and CSF biomarkers in brain subcortical ischemic vascular disease: involved pathways and clinical applicability. *J. Cereb. Blood Flow Metab.* 36 55–71. 10.1038/jcbfm.2015.68 25899297 PMC4758557

[B46] Wierenga C. E. Hays C. C. Zlatar Z. Z. (2014). Cerebral blood flow measured by arterial spin labeling MRI as a preclinical marker of Alzheimer’s disease. *J. Alzheimers. Dis.* 42 (Suppl. 4), S411–S419. 10.3233/jad-141467 25159672 PMC5279221

[B47] Wu X. Ge X. Du J. Wang Y. Sun Y. Han X. (2019). Characterizing the penumbras of white matter hyperintensities and their associations with cognitive function in patients with subcortical vascular mild cognitive impairment. *Front. Neurol.* 10:348. 10.3389/fneur.2019.00348 31031687 PMC6474292

[B48] Xin S. H. Tan L. Cao X. Yu J. T. Tan L. (2018). Clearance of amyloid beta and tau in Alzheimer’s disease: from mechanisms to therapy. *Neurotox. Res.* 34 733–748. 10.1007/s12640-018-9895-9891 29626319

[B49] Xu G. Rowley H. A. Wu G. Alsop D. C. Shankaranarayanan A. Dowling M. (2010). Reliability and precision of pseudo-continuous arterial spin labeling perfusion MRI on 3.0 T and comparison with 15O-water PET in elderly subjects at risk for Alzheimer’s disease. *NMR Biomed.* 23 286–293. 10.1002/nbm.1462 19953503 PMC2843795

[B50] Yamada M. (2015). Cerebral amyloid angiopathy: emerging concepts. *J. Stroke* 17 17–30. 10.5853/jos.2015.17.1.17 25692104 PMC4325636

[B51] Yew B. Nation D. A. (2017). Cerebrovascular resistance: effects on cognitive decline, cortical atrophy, and progression to dementia. *Brain* 140 1987–2001. 10.1093/brain/awx112 28575149 PMC6059092

[B52] Zhu J. Zhuo C. Qin W. Xu Y. Xu L. Liu X. (2015). Altered resting-state cerebral blood flow and its connectivity in schizophrenia. *J. Psychiatr. Res.* 63 28–35. 10.1016/j.jpsychires.2015.03.002 25812945

[B53] Zhu J. Zhuo C. Xu L. Liu F. Qin W. Yu C. (2017). Altered coupling between resting-state cerebral blood flow and functional connectivity in schizophrenia. *Schizophr. Bull.* 43 1363–1374. 10.1093/schbul/sbx051 28521048 PMC5737873

