# Supplementary material for: Altered serum amyloid beta and cerebral perfusion and their associations with cognitive function in patients with subcortical ischemic vascular disease
Source: Front Neurosci. 2022 Oct 13;16:993767. doi: 10.3389/fnins.2022.993767 (PMC9608371; doi:10.3389/fnins.2022.993767)
Supplement: Supplementary file 1 [file Data_Sheet_1.pdf]

Altered serum amyloid beta and cerebral perfusion and their associations with cognitive function in patients with subcortical ischemic vascular disease

Figure S1 | Scatter plots of correlation between serum Aβ40/42 ratio, WMHV, and CBF (including whole brain, GM, and WM) in patients with the whole SIVD

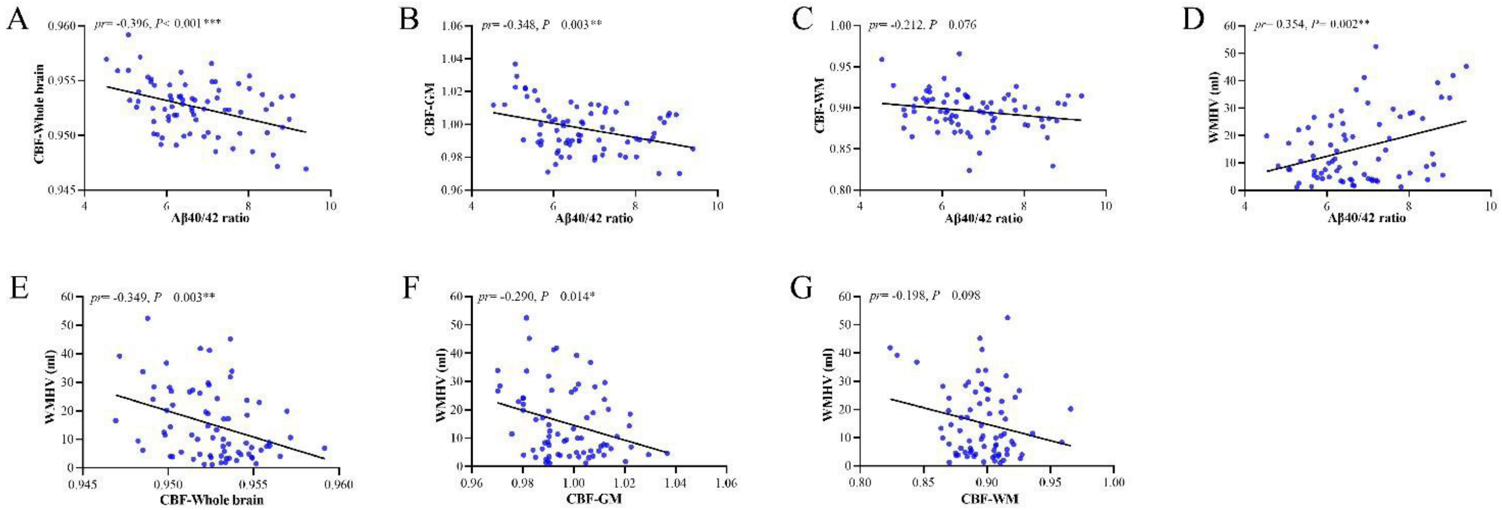

(A) Negative correlations between serum Aβ40/42 ratio and CBF in the whole brain. **(B)** Negative correlations between serum Aβ40/42 ratio and CBF in GM. **(C)** Negative correlations between serum Aβ40/42 ratio and CBF in WM. **(D)** Positive correlations between serum Aβ40/42 ratio and WMHV. **(E)** Negative correlations between CBF in the whole brain and WMHV. **(F)** Negative correlations between CBF in the GM and WMHV. **(G)** Negative correlations between CBF in the WM and WMHV. pr, partial correlation coefficient; CBF, cerebral blood flow; GM, gray matter; WM, white matter; WMHV, white matter hyperintensity volume *P <0.05; **P <0.01; ***P <0.01. (controlling for age, gender, education, vascular risk factors).

Figure S2 | Scatter plots of correlation between CBF, WMHV, and neuropsychological tests in patients with the whlole SIVD

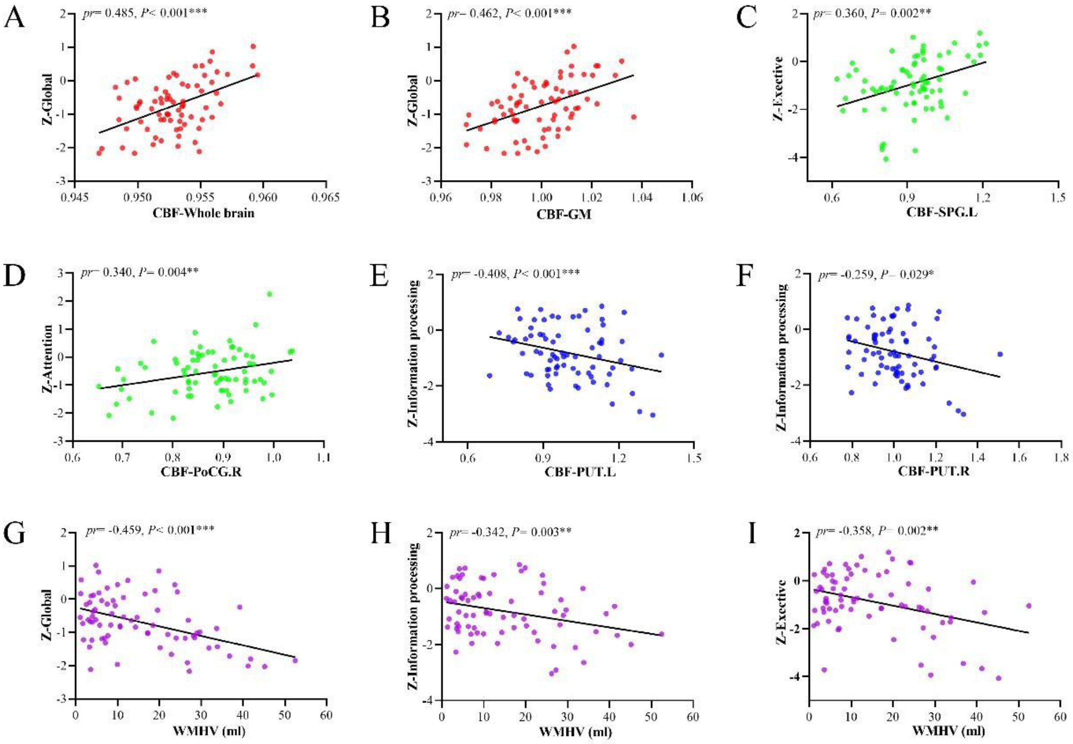

(A) Positive correlations between CBF in the whole brain and z-scores in global. **(B)** Positive correlations between CBF in the GM and z-scores in global. **(C)** Positive correlations between CBF in the SPG.L and z-scores in exective function. **(D)** Positive correlations between CBF in the PoCG.R and z-scores in attention function. **(E)** Negative correlations between CBF in PUT.L and z-scores in information processing function. **(F)** Negative correlations between CBF in PUT.R and z-scores in information processing function. **(G)** Negative correlations between WMHV and z-scores in global cognition. **(H)** Negative correlations between WMHV and z-scores in information processing function. **(I)** Negative correlations between WMHV and z-scores in executive function. CBF, cerebral blood flow; pr, partial correlation coefficient; GM, gray matter; SPG.L, left superior parietal gyrus; PoCG.R, right postcentral gyrus; PUT, putamen; WMHV, white matter hyperintensity volume; L, left; R, right. **P* <0.05; ***P* <0.01; ****P* <0.001. (controlling for age, gender, education, vascular risk factors).

Figure S3 | Conceptual diagram of mediation analysis between Aβ40/42 ratio, CBF, and global cognition in patients with the whole SIVD

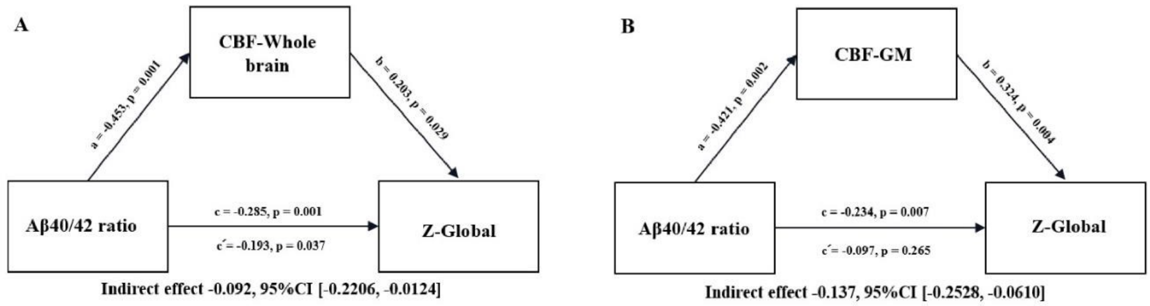

(A) serum Aβ40/42 ratio (X), CBF in the whole brain (M), z-score of global (Y). **(B)** serum Aβ40/42 ratio (X), CBF in the GM (M), z-score of global (Y). Path coefficients with *P* values. GM, gray matter; CBF, cerebral blood flow; CI, confidence interval.

Stratified analysis

Table S1 Demographic, clinical, neuropsychological tests, and neuroimaging manifestations data

Characteristics	**HC** **(age=50-65)** (n=42)	**SIVD** **(age=50-65)** (n=36)	*P*	**HC** **(age=66-80)** (n=32)	**SIVD** **(age=66-80)** (n=38)	*P*
**Demographics**						
Age (years)	56.45 (4.32)	57.50 (4.76)	0.312 [b]	69.75 (3.14)	70.79 (3.46)	0.197 [b]
Male, n (%)	16 (38.1)	17 (47.2)	0.416 [a]	14 (43.8)	25 (65.8)	0.064 [a]
Education (years)	9.33 (2.73)	8.47 (2.71)	0.167 [b]	8.50 (3.02)	8.92 (3.02)	0.564 [b]
**Vascular risk factors**						
Hypertension, n (%)	5 (11.9)	18 (50.0)	**<0.001** [a]	5 (15.6)	19 (50.0)	**<0.001** [a]
Diabetes, n (%)	4 (9.5)	12 (33.3)	**0.009** [a]	4 (12.5)	8 (21.1)	0.344 [a]
Hyperlipidemia, n (%)	3 (7.1)	14 (38.9)	**<0.001** [a]	7 (21.9)	13 (34.2)	0.255 [a]
Smoking history, n (%)	2 (4.8)	7 (19.4)	0.073 [a]	7 (21.9)	11 (28.9)	0.500 [a]
Drinking history, n (%)	3 (7.1)	12 (33.3)	**0.003** [a]	6 (18.8)	16 (42.1)	**0.036** [a]
**Laboratory examination**						
Glu (mmol/L)	5.38 (0.74)	5.76 (1.42)	0.138 [b]	5.42 (1.60)	5.62 (0.99)	0.519 [b]
TG (mmol/L)	1.41 (0.66)	1.49 (1.03)	0.677 [b]	1.51 (0.96)	1.45 (1.01)	0.784 [b]
TCH (mmol/L)	4.36 (0.64)	4.80 (1.31)	0.059 [b]	4.12 (0.87)	4.11 (1.11)	0.965 [b]
LDL-C (mmol/L)	3.13 (0.67)	2.88 (1.14)	0.235 [b]	2.89 (0.76)	2.84 (0.88)	0.897 [b]
HDL-C (mmol/L)	1.48 (0.41)	1.36 (0.32)	0.182 [b]	1.44 (0.34)	1.37 (0.47)	0.508 [b]
Hcy (umol/L)	15.55 (3.44)	19.14 (9.93)	**0.031** [b]	17.90 (6.43)	19.70 (10.41)	0.400 [b]
hs-CRP (mg/L)	1.73 (2.44)	1.91 (2.81)	0.768 [b]	1.51 (2.27)	2.31 (3.52)	0.277 [b]
Aβ40 (pg/mL)	399.78 (28.91)	410.84 (26.05)	0.082 [b]	398.11 (32.50)	420.65 (38.12)	**0.010** [b]
Aβ42 (pg/mL)	66.80 (9.05)	62.65 (10.04)	0.059 [b]	66.58 (9.70)	62.34 (8.67)	0.058 [b]
Aβ40/42 ratio	6.08 (0.86)	6.71 (1.12)	**0.006** [b]	6.13 (1.17)	6.88 (1.18)	**0.010** [b]
APOE4, n (%)	8 (19.0)	7 (19.4)	0.965 [a]	3 (9.4)	12 (31.6)	**0.024** [a]
**Neuropsychological tests**						
Z-Information processing	0.0 (0.75)	-0.53 (0.81)	**0.004** [b]	0.0 (0.73)	-1.06 (0.95)	**<0.001** [b]
Z-Executive	0.0 (0.79)	-0.64 (1.10)	**0.004** [b]	0.0 (0.71)	-1.08 (1.29)	**<0.001** [b]
Z-Attention	0.0 (0.89)	-0.53 (0.71)	**0.005** [b]	0.0 (0.89)	-0.55 (0.84)	**0.009** [b]
Z-Memory	0.0 (0.82)	-0.41 (1.04)	0.057 [b]	0.0 (0.87)	-0.85 (1.01)	**<0.001** [b]
Z-Language	0.0 (0.76)	-0.37 (0.93)	0.053 [b]	0.0 (0.88)	-0.63 (0.87)	**0.004** [b]
Z-Visuospatial	0.0 (1.00)	-0.85 (1.53)	**0.004** [b]	0.0 (1.00)	-1.00 (1.47)	**0.002** [b]
Z-Global	0.0 (0.47)	-0.49 (0.69)	**<0.001** [b]	0.0 (0.60)	-0.85 (0.81)	**<0.001** [b]
**Neuroimaging**						
GMV (mL)	585.64 (41.78)	594.13 (51.35)	0.463 [b]	575.03 (49.67)	562.75 (54.82)	0.334 [b]
WMV (mL)	471.88 (56.13)	481.82 (54.28)	0.431 [b]	467.88 (54.89)	451.57 (49.28)	0.195 [b]
WMHV	0.93 (0.67)	7.61 (21.38)	**<0.001** [c]	1.19 (1.00)	14.01 (17.20)	**<0.001** [c]
Lacunes	0 (0)	0 (1)	0.136 [c]	0 (1)	0.5 (1)	0.088 [c]
CBF-Whole brain	0.9547 (0.0025)	0.9533 (0.0029)	**0.031** [b]	0.9553 (0.0025)	0.9533 (0.0026)	**0.003** [b]
CBF-GM	1.0072 (0.0128)	1.0004 (0.0132)	**0.025** [b]	1.0047 (0.0128)	0.9971 (0.0136)	**0.020** [b]
CBF-WM	0.9025 (0.0175)	0.8946 (0.0286)	0.141 [b]	0.9038 (0.0201)	0.9001 (0.0248)	0.500 [b]

Note: Continuous variables with normal distribution (age, Glu, TG, TCH, LDL-C, HDL-C, Hcy, hs-CRP, Aβ40, Aβ42, Aβ40/42 ratio, Neuropsychological tests, GMV, WMV, CBF-Whole brain, CBF-GM, CBF-WM) were described by mean and standard deviation. Continuous variables with non-normal distribution (WMHV, Lacunes) were described by median and interquartile range. Categorical variables (sex, hypertension diabetes, hyperlipidemia, smoking history, drinking history, APOE4) were described by frequencies and percentages. a: Chi-square test; b: Two independent-samples t test; c: Mann-Whitney U test; HC: healthy control; SIVD: subcortical ischemic vascular disease; Glu: glucose; TCH: total cholesterol; TG: triglycerides; HDL-C: high density lipoprotein-cholesterol; LDL-C: low density lipoprotein-cholesterol; Hcy: homocysteine; hs-CRP: hypersensitive-C reactive protein; GMV: gray matter volume; WMV: white matter volume; WMHV: white matter hyperintensity volume; GM: gray matter; WM: white matter; CBF: cerebral blood flow.

Figure S4 | ROC analysis of the serum Aβ40, Aβ42, and Aβ40/42 levels in the diagnosis of the SIVD and HCs

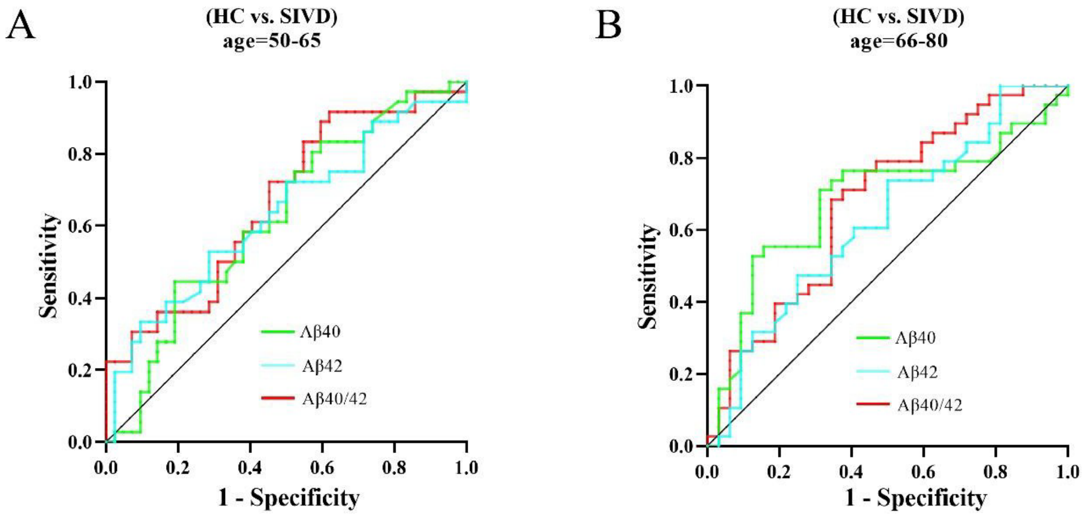

(A) ROC analyses of serum Aβ40, Aβ42, and Aβ40/42 levels for SIVD patients aged 50-60 years versus HCs aged 50-60 years. **(B)** ROC analyses of serum Aβ40, Aβ42, and Aβ40/42 levels for SIVD patients aged 66-80 years versus HCs aged 66-80 years. ROC, receiver operating characteristic; HC, healthy control; SIVD, subcortical ischemic vascular disease; vs, versus.

Figure S5 | Scatter plots of correlation between serum Aβ40/42 ratio, WMHV, and CBF (including whole brain, GM, and WM) in patients with SIVD (age = 50-65 and age = 66-80).

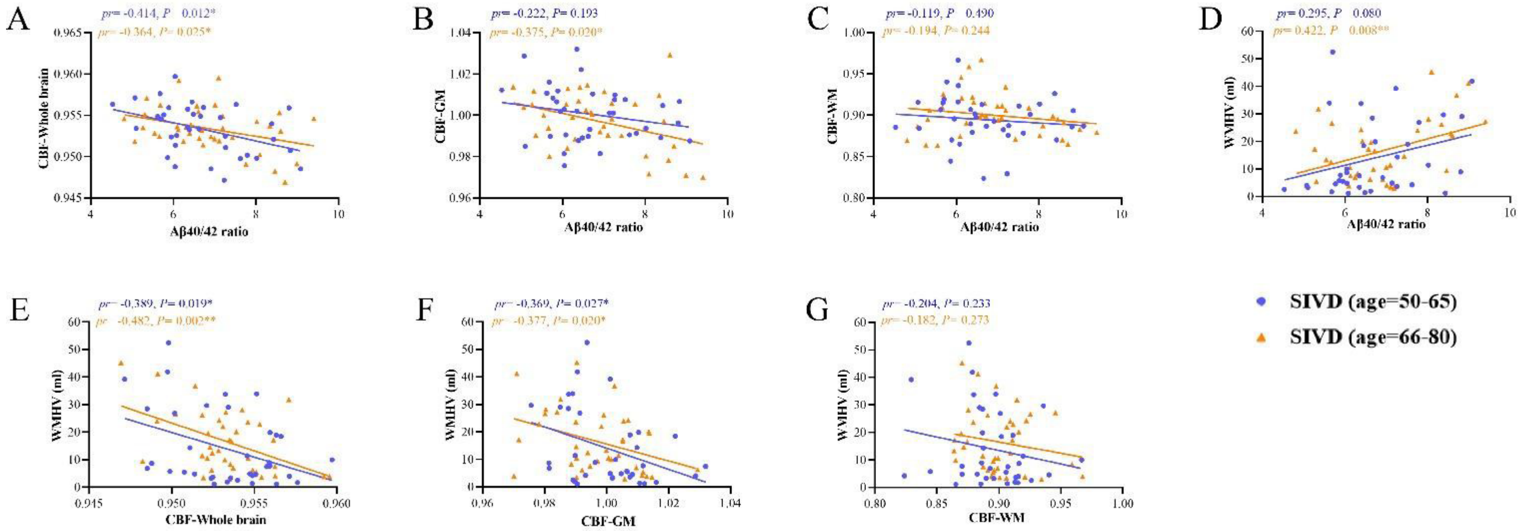

The blue dots represent the SIVD group aged 50-65, and the orange-yellow triangle represents the SIVD group aged 66-80. (A) Negative correlations between serum Aβ40/42 ratio and CBF in the whole brain. **(B)** Negative correlations between serum Aβ40/42 ratio and CBF in GM. **(C)** Negative correlations between serum Aβ40/42 ratio and CBF in WM. **(D)** Positive correlations between serum Aβ40/42 ratio and WMHV. **(E)** Negative correlations between CBF in the whole brain and WMHV. **(F)** Negative correlations between CBF in the GM and WMHV. **(G)** Negative correlations between CBF in the WM and WMHV. pr, partial correlation coefficient; CBF, cerebral blood flow; GM, gray matter; WM, white matter; WMHV, white matter hyperintensity volume. **P* <0.05; ***P* <0.01; ****P* <0.01. (controlling for age, gender, education, vascular risk factors).

Figure S6 | Scatter plots of correlation between CBF, WMHV, and neuropsychological tests in patients with SIVD (age = 50-65 and age = 66-80)

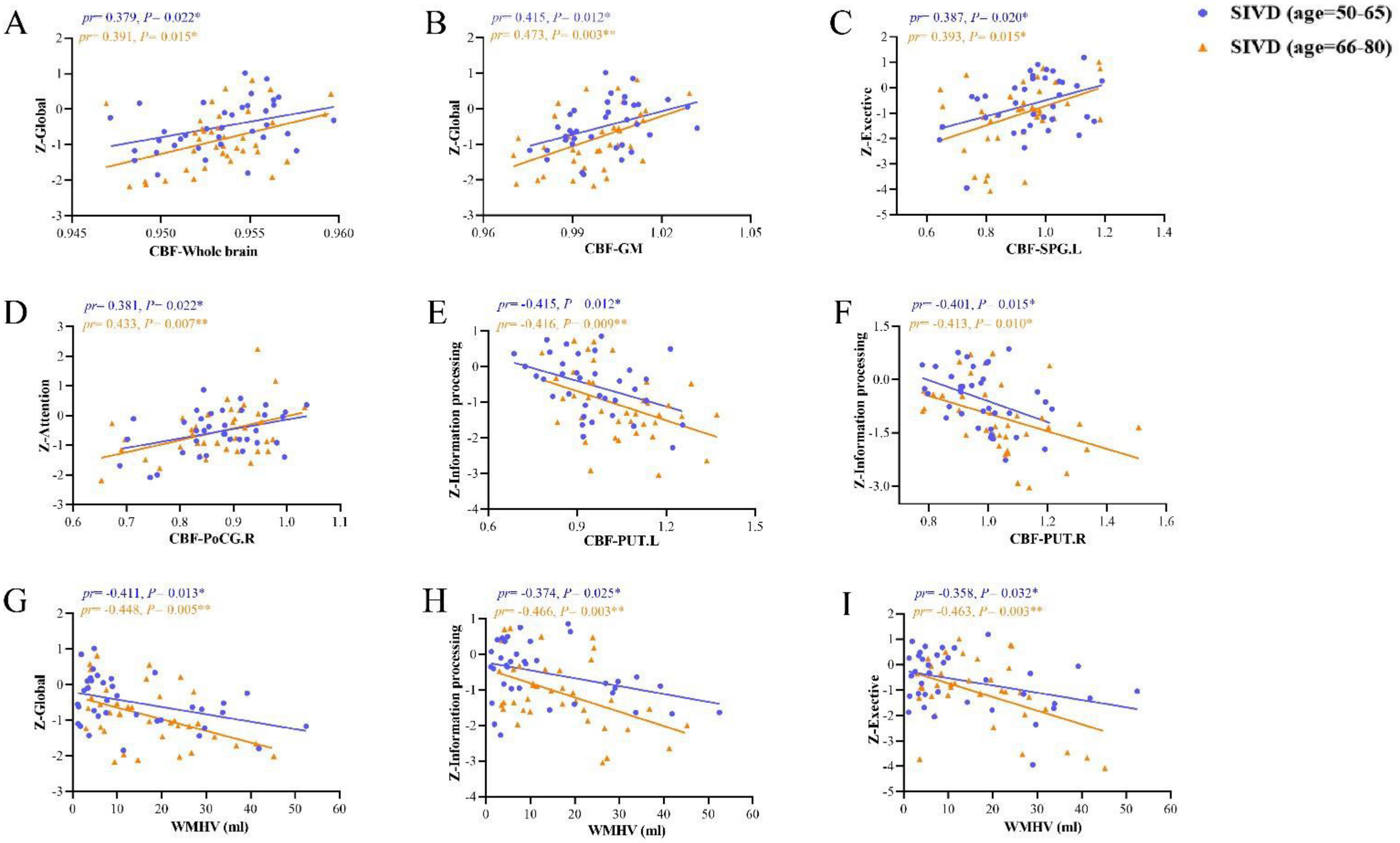

The blue dots represent the SIVD group aged 50-65, and the orange-yellow triangle represents the SIVD group aged 66-80. (A) Positive correlations between CBF in the whole brain and z-scores in global. **(B)** Positive correlations between CBF in the GM and z-scores in global. **(C)** Positive correlations between CBF in the SPG.L and z-scores in exective function. **(D)** Positive correlations between CBF in the PoCG.R and z-scores in attention function. **(E)** Negative correlations between CBF in PUT.L and z-scores in information processing function. **(F)** Negative correlations between CBF in PUT.R and z-scores in information processing function. **(G)** Negative correlations between WMHV and z-scores in global cognition. **(H)** Negative correlations between WMHV and z-scores in information processing function. **(I)** Negative correlations between WMHV and z-scores in executive function. CBF, cerebral blood flow; pr, partial correlation coefficient; GM, gray matter; SPG.L, left superior parietal gyrus; PoCG.R, right postcentral gyrus; PUT, putamen; WMHV, white matter hyperintensity volume; L, left; R, right. **P* <0.05; ***P* <0.01; ****P* <0.001. (controlling for age, gender, education, vascular risk factors).

Figure S7 | Conceptual diagram of mediation analysis between Aβ40/42 ratio, CBF, and global cognition in patients with SIVD (age = 50-65 and age = 66-80)

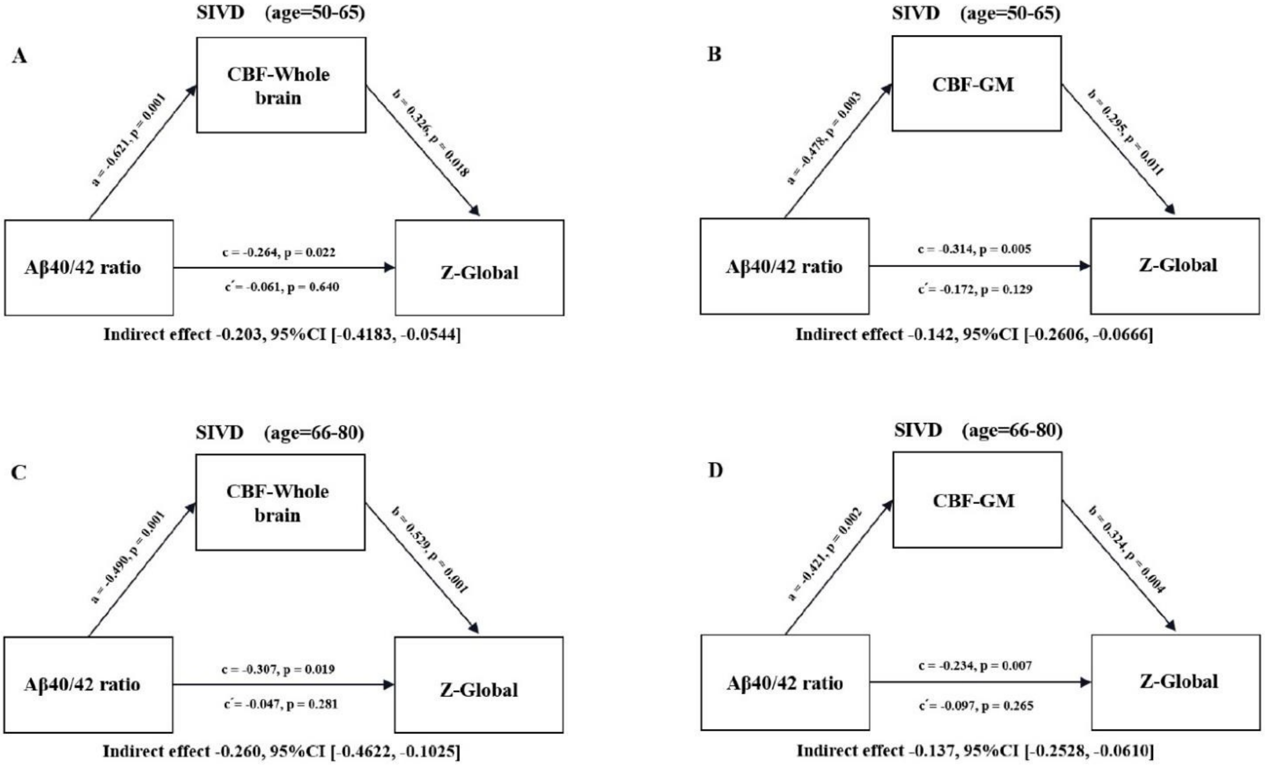

Conceptual diagram of a mediation analysis model with one mediator. Total effect of X on Y (c) = indirect effect of X on Y through M (a × b) + direct effec t of X on Y (c’). **(A)** serum Aβ40/42 ratio (X), CBF in the whole brain (M), z-score of global (Y) in patients with SIVD aged 50-65 years. **(B)** serum Aβ40/42 ratio (X), CBF in the GM (M), z-score of global (Y) in patients with SIVD aged 50-65 years. **(C)** serum Aβ40/42 ratio (X), CBF in the whole brain (M), z-score of global (Y) in patients with SIVD aged 66-80 years. **(D)** serum Aβ40/42 ratio (X), CBF in the GM (M), z-score of global (Y) in patients with SIVD aged 66-80 years. Path coefficients with *P* values. GM, gray matter; CBF, cerebral blood flow; CI, confidence interval.
